# Ring strain and total syntheses of modified macrocycles of the isoplagiochin type

**DOI:** 10.3762/bjoc.5.71

**Published:** 2009-12-01

**Authors:** Andreas Speicher, Timo Backes, Kerstin Hesidens, Jürgen Kolz

**Affiliations:** 1 Department 8.1 Chemistry – Organic Chemistry, Saarland University, 66041 Saarbrücken, Germany

**Keywords:** axially chiral biaryl, isoplagiochin, macrocycle, ring strain, total synthesis

## Abstract

Macrocycles of the bisbibenzyl-type are natural products that are found exclusively in bryophytes (liverworts). The molecular framework of the subtype “isoplagiochin” is of substantial structural interest because of the chirality of the entire molecule, which arises from two biaryl axes in combination with two helical two-carbon units in a cyclic arrangement. From a structural as well as a synthetic point of view we report on the total synthesis of compounds which possess more rigid two-carbon biaryl bridges like stilbene (*E* or *Z*) or even tolane moieties which were introduced starting with a Sonogashira protocol. The McMurry method proved to be a powerful tool for the cyclization to these considerably ring-strained macrocycles.

## Introduction

**Figure 1:**
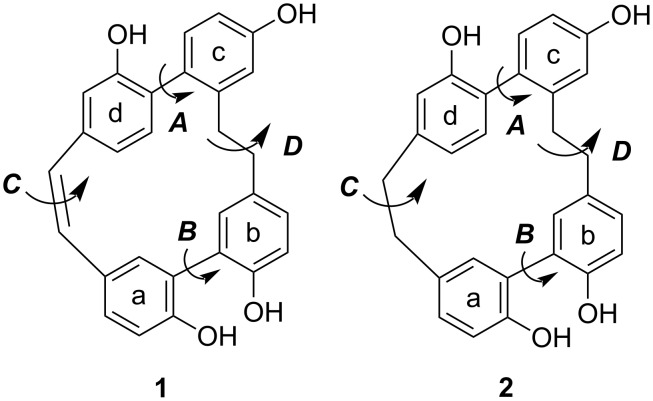
Structures of isoplagiochins C (**1**) and D (**2**) (with aryl fragments a–d and possible conformational barriers ***A***–***D***).

The cyclic bisbibenzyls isoplagiochin C (**1**) and D (**2**) were isolated from the liverworts *Plagiochila fruticosa* [1], *Plagiochila deflexa* [2], Herbertus sakuraii [3] and *Lepidozia fauriana* [4] (Figure 1). Recently, an increasing number of different biological activities of phenolic compounds of the bisbibenzyl type were reported [5–12].

**Figure 2:**
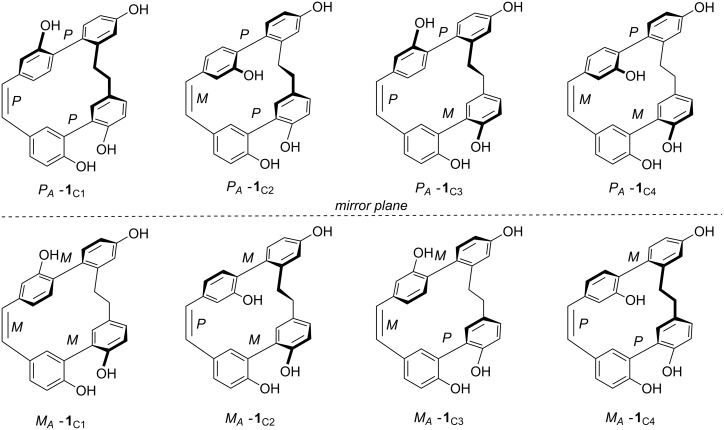
Possible stereoisomers of **1** as conformers C1–C4 relative to the configurationally stable biaryl axis ***A*** with the stereo descriptors *P* or *M*.

Furthermore, the isoplagiochin framework proved to be of substantial structural interest because of the chirality of the entire molecule. The existence of atropoisomers [3] prompted us to perform detailed studies on the chirality of **1** and **2** including HPLC-CD experiments. Assuming the existence of three formal stereogenic elements ***A***–***C*** (Figure 1), a total of up to 2^3^ = 8 conformers (four diastereomers with their enantiomers) in principle are possible for **1** (and analogously for **2**) (Figure 2). The ethylene bridge ***D*** is more flexible in these two molecules. Temperature dependent NMR investigations did not provide any evidence for the existence of different diastereomers configurationally stable within the NMR timescale. AM1-based conformational analysis and MD investigations clearly confirmed the biaryl axis ***A*** to be configurationally stable at room temperature due to the second (more flexible) biaryl axis ***B***, an (even more flexible) helical stilbene unit ***C*** and their combination with the ring-strain of the entire molecule.

**Scheme 1:**
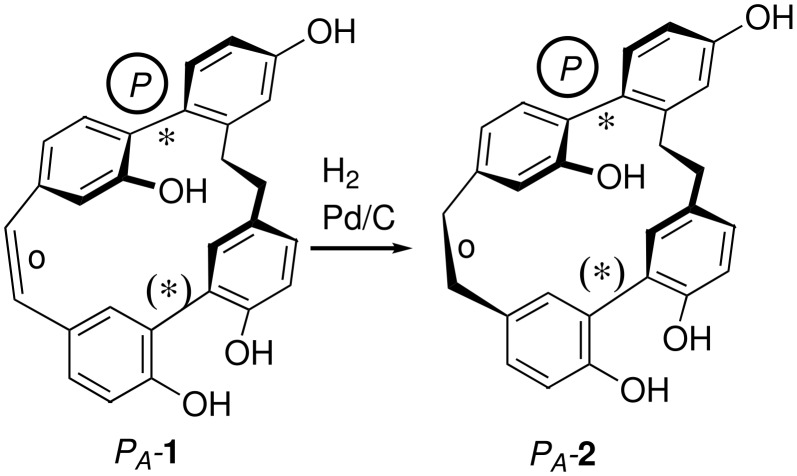
Stereochemical correlation for **1** and **2**. (*: configurationally stable, (*): configurationally semi-stable; o: configurationally unstable).

By experimental and quantum chemical CD (circular dichroism) investigations, the absolute configuration of the first natural compound of this type, isoplagiochin C from *P. deflexa*, was established as (*P_A_*)-**1** and the energy of racemization was calculated and measured to be 102 kJ/mol, approximately [13]. The enantiomers (*P_A_*)-**1** and (*M_A_*)-**1** are each manifested in the four imaginable, rapidly interconverting diastereomers whereby the C2 conformations were estimated as the global minima (Figure 2). Similar experiments and calculations were performed for isoplagiochin D (**2**) and for some chlorinated derivatives. The stereochemical correlation between isoplagiochin C (**1**) and isoplagiochin D (**2**) was also confirmed experimentally by hydrogenolysis of enantiopure samples of (*P_A_*)-**1** to give and (*P_A_*)-**2** (Scheme 1), and of (*M_A_*)-**1** to give and (*M_A_*)-**2**, respectively, without any racemization [14].

The natural compounds isoplagiochin D (**2**) and C (**1**) possess two saturated ethylene bridges (between rings **a**–**d** and **b**–**c**) or just one *cis*-configurated double bond (’stilbene bridge’) between rings **a**–**d**. From a structural as well as a synthetic point of view we were interested in compounds which possess various unsaturated stilbene (*E* or *Z*) or even tolane bridges between the two biaryl units **a**–**b** and **c**–**d**. These structures should be more rigid with respect to the ring strain enhanced by geometrically fixed two-carbon bridges. But for ring strain considerations, the *real* cyclization product possessing at least additional phenolic protecting groups should be studied.

## Results and Discussion

**Figure 3:**
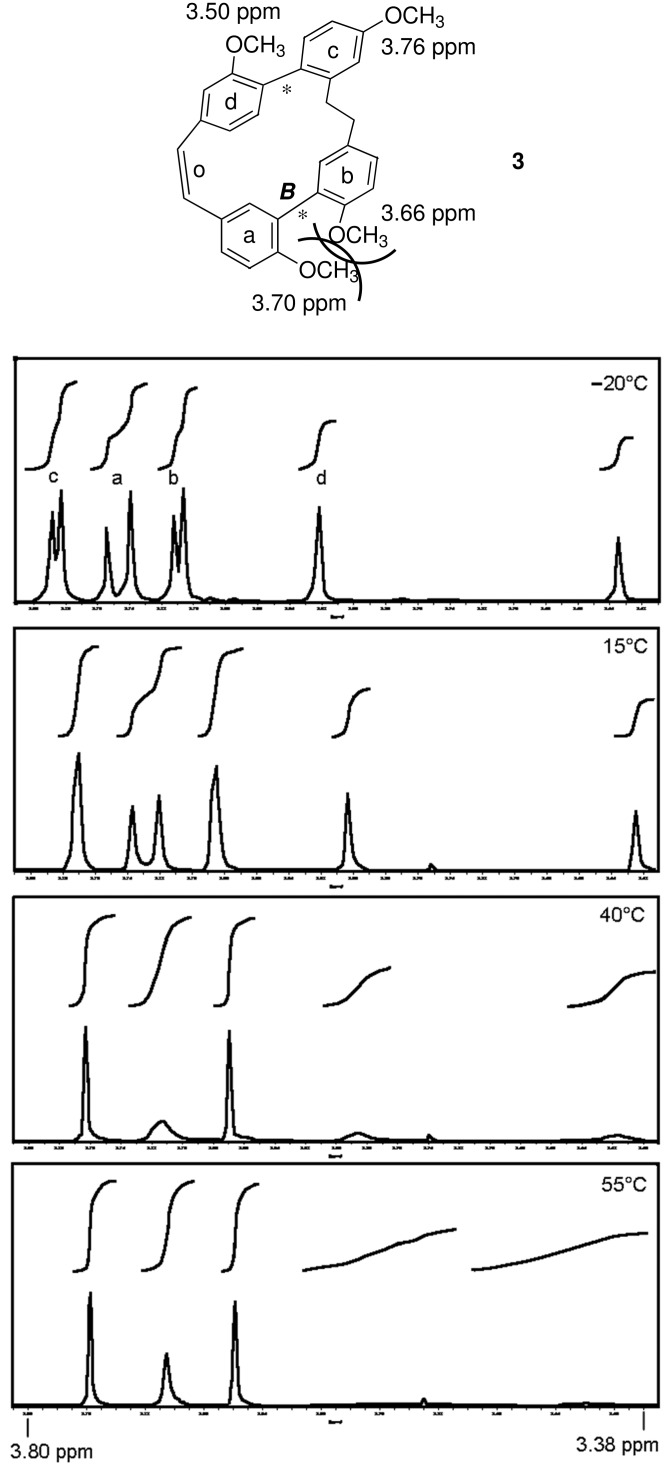
Temperature dependent ^1^H NMR and assignment of methoxy signals in the tetramethyl ether **3**.

In our total syntheses of the racemic natural compounds **1** and **2** reported previously [15] the common and crucial precursor was the tetramethyl ether **3**. Surprisingly and in contrast to the NMR results mentioned above for **1**, closer NMR examination now revealed two sets of signals for **3** indicating two conformers (ratio 1.3 : 1) stable within the NMR time scale. This can be interpreted as due to a much less flexible axis ***B*** resulting from the stereochemically more demanding methoxy groups in the *o,o*′-positions (Figure 3).

Indeed, temperature dependent ^1^H NMR investigations from −20 up to 55 °C resulted in coalescence of the methoxy signals. By an approximation method [16] as well as by exact line form analysis [17] the rotation barrier could be calculated to ~66 kJ/mol indicating that the conformers are stable within the NMR time scale but cannot be separated. By 2D NMR experiments (COSY, HSQC, HMBC) at 100 °C four sharp signals could be assigned to the methoxy groups in rings **a**–**d** (Figure 3).

To gain insight into possible ring strain limitations for the preparation and stability of modified macrocycles of the isoplagiochin type we performed molecular mechanic and semi-empirical calculations for tetramethyl protected target compounds. Together with the molecules “in hand” they clearly demonstrated that the bridge between **a**–**d** should be saturated or at least a *Z* double bond as in the natural compounds **1** (cf. **3**) and **2** (cf. **4**). An *E* double bond or an alkyne moiety are not well tolerated (see e.g. **8**, **9**), even in the system with the lowest possible strain, a saturated **b**–**c**-bridge. This can be deduced mainly from the substantial angular strain in the alkyne moiety (see **8**) or from the distortion in the (*E*)-alkene moiety (see **9**) as well as in torsion stress looking at the aromatic rings **a** and **d** in both candidates. The obvious reason for these effects is the *para*-insertion of the aryl moiety **d** into the macrocyclic system. In contrast, for the bridge **b**–**c**, the *Z* and *E* double bond as well as a tolane bridge should be more easily realizable with respect to angular strain and torsion stress (see compounds **5**–**7** in Table 1).

**Table 1:** AM1 energies [18], angular strain and torsion stress of different modified macrocycles of the isoplagiochin type.

Compound	Molecular energy^a^ and ring-strain	Compound	Molecular energy^a^ and ring-strain

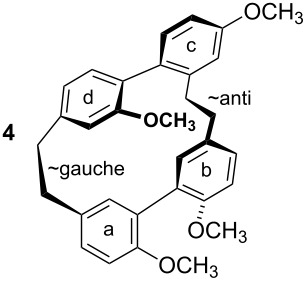	−7432 no torsion **low**	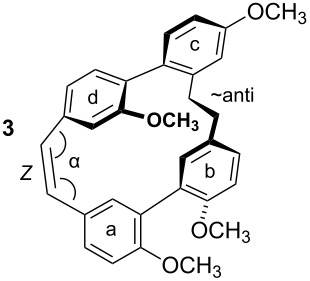	−7300 α ~126° no torsion **low**
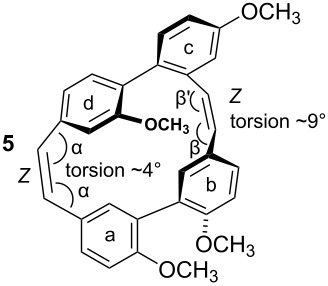	−7160 α = 128° β = 130° β′ = 132° **moderate**	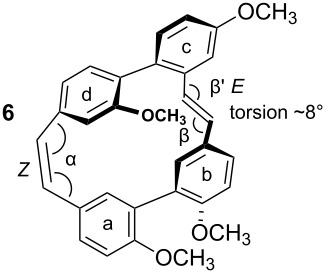	−7167 α ~129° β = 119° β′ = 128° **moderate**
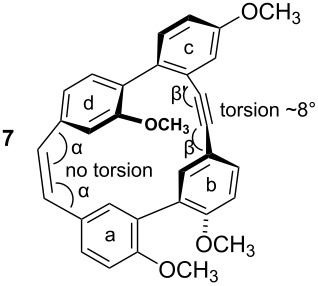	−7025 α = 129° β = 168° β′ = 175° **moderate**	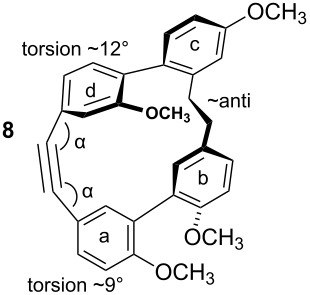	−7130 α = 155° **high**
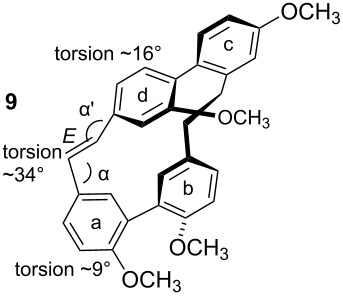	−7274 α = 120° α′ = 123° **high**		

^a^The calculations are given for the conformations resembling the most stable *P_A_*-**1**_C2_ conformer (see Figure 1). No significant deviations were observed for other conformers. Note that molecular energy values should only be compared for isomeric compounds.

As a consequence of these results we were interested in the syntheses of the new bisbibenzyl frameworks **5**–**7**. Our previously reported total syntheses of the racemic natural compounds **1** and **2** [15,19] or some derivatives [20] could not be adopted because they do not give rise to specific double bond geometry or even an alkyne moiety between rings **b** and **c**.

**Scheme 2:**
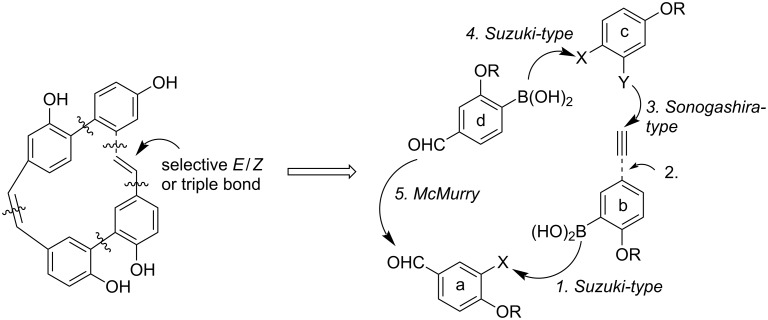
Strategy of synthesis for the macrocycles **5**–**7**.

Our new strategy of synthesis involved a consecutive coupling of the rings **a-b-c-d** whereby the two-carbon bridge **b**–**c** would be introduced by a Sonogashira type reaction yielding an alkyne moiety (see **7**), from which a *Z* double bond (for **5**) or an *E* double bond may be obtainable by Lindlar-type hydrogenation and (for **6**) by subsequent *Z* → *E* isomerization (Scheme 2). The final ring closure using a McMurry protocol (probably more rigorous for ring-strained frameworks than the Wittig-protocol in the syntheses of **3**/**4**) should give rise only to the *Z* geometry at the **a**–**d** stilbene bridge.

**Scheme 3:**
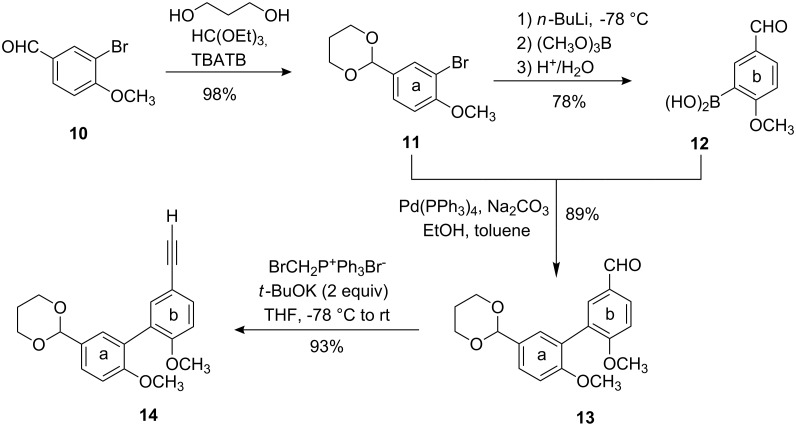
Preparation of the terminal alkyne **13** as **a**–**b** part (TBATB = tetrabutylammonium tribromide).

The **a**–**b** part **14** [15] of the target molecules was prepared by Suzuki coupling of the boronic acid **12** with its precursor **11**, readily available from 3-bromo-4-methoxybenzaldehyde (**10**) [21] by an improved procedure [22]. The aldehyde **13** was transformed into the terminal alkyne **14** by chain extension [23] (Scheme 3).

**Scheme 4:**
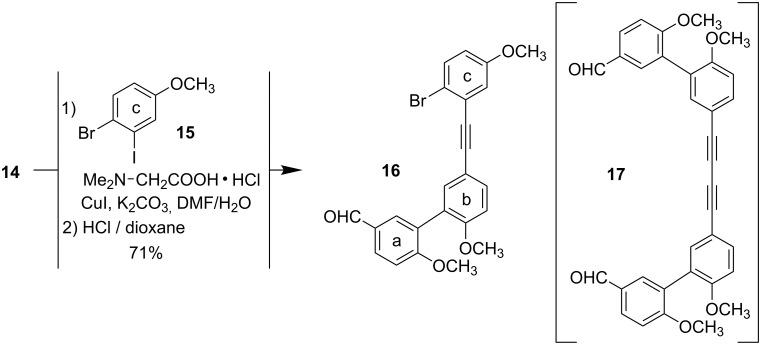
Sonogashira-type coupling to the tolane **16**.

The coupling of the terminal alkyne **14** with the iodoarene **15** [24] as the “**c**” building block using a typical Sonogashira protocol [Pd(PPh_3_)_4_, CuI, Et_3_N] gave – even under an argon–hydrogen atmosphere [25] – only 30% yield beside the undesired homo-coupling product **17**. A modified and palladium free procedure, however [26], resulted – after acidic workup liberating the aldehyde group – in a satisfactory yield of the mixed tolane **16** as the **a-b-c** precursor (Scheme 4).

**Scheme 5:**
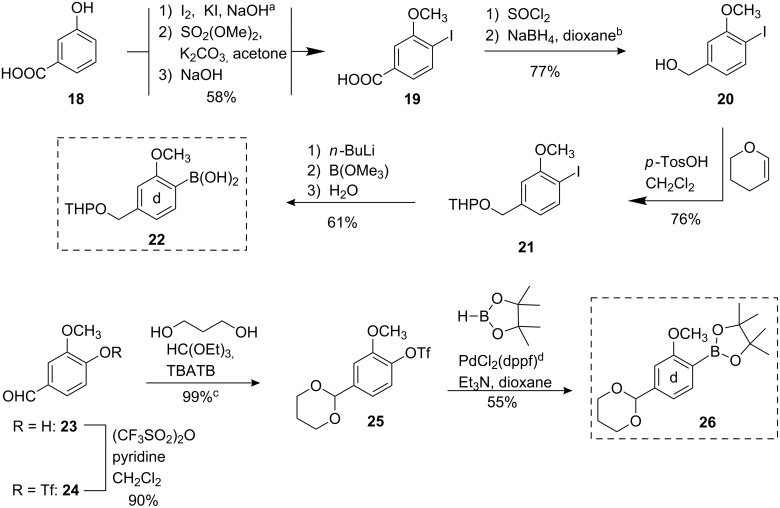
Synthesis of the **d** building blocks **21** and **26**. ^a^The original procedure in diluted ammonia [31] was replaced because deflagration to detonation was observed in some cases due to formation of nitrogen iodide (NI_3_). ^b^Attempts to direct reduction of the methyl ester or the carboxylic acid using e. g. LiAlH_4_ failed due to concomitant deiodination. ^c^Protection of the aldehyde was obligatory to prevent exclusive pinacolborane reduction. ^d^dppf: 1,1′-bis(diphenylphosphino)ferrocene.

Two different building blocks **22** and **26** were prepared as the “**d**” ring for Suzuki coupling with the bromoarene **16** (Scheme 5). First, iodination of 3-hydroxybenzoic acid (**18**) followed by double methylation and saponification of the methyl ester yielded 4-iodo-3-methoxybenzoic acid (**19**) which could be reduced in two steps [27] to the benzylic alcohol **20** [28]. Protection as the THP acetal **21** was essential before preparing the boronic acid **22**. Previously (see [15]), we reported on a synthesis of **22** starting with the bromination of 3-methoxybenzyl alcohol to 4-bromo-3-methoxybenzyl alcohol. In contrast to the original contribution [29] bromination lead to the formation of 2-bromo-5-methoxybenzyl alcohol, exclusively.

Second, the triflate **24** was obtained from vanillin (**23**) and was further dioxane-protected at the aldehyde function (to **25**) and directly transformed into the arylboronate **26** using pinacolborane [30].

**Scheme 6:**
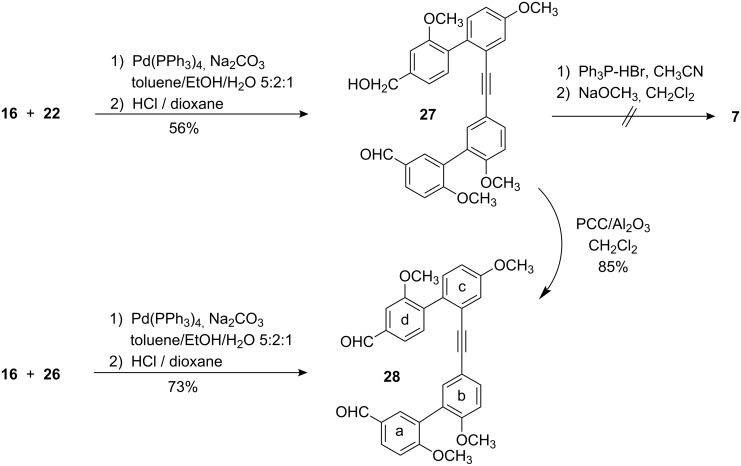
Synthesis of the tolane precursors **27** and **28** for cyclization.

Suzuki coupling of the boronic acid **22** with the bromoarene **16** under carefully optimized conditions followed by acidic workup afforded the hydroxyaldehyde **27** as precursor for cyclization (Scheme 6). A cyclization protocol for the intramolecular Wittig reaction, well established [15,20] for the synthesis of the tetramethyl ether of isoplagiochin C (**1**), however failed in this case. This might be due to the enhanced ring strain. Since the McMurry reaction would provide more rigorous conditions for the formation of ring-strained frameworks, the hydroxyaldehyde **27** was oxidized to the dialdehyde **28**. It is worth mentioning that **28** could be obtained more straightforwardly and in higher overall yield directly from the bromoarene **16** and the arylboronate **26**.

**Scheme 7:**
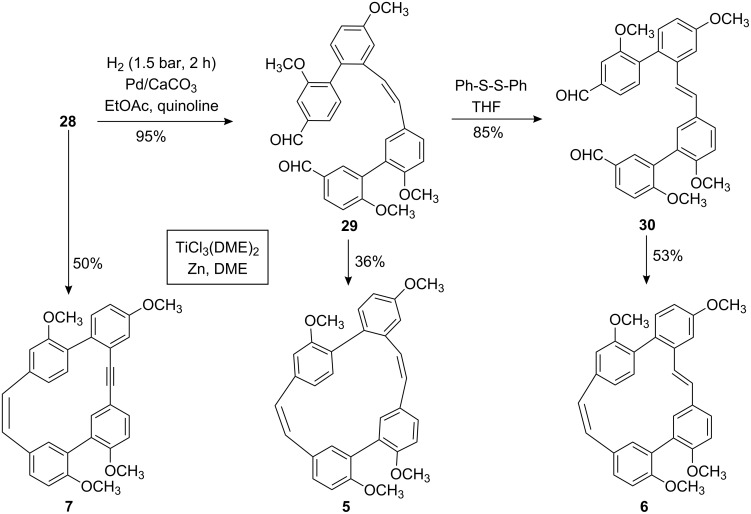
Synthesis of the modified macrocycles **5**–**7** from the dialdehyde precursors **28**–**30**.

Indeed, the cyclization of **28** to the macrocycle **7** bearing an “alkyne bridge” was successful in moderate yield using a McMurry protocol (Scheme 7) [32]. Hydrogenation of the tolane **28** using Lindlar conditions selectively afforded the (*Z*)-alkene **29** from which the (*E*)-alkene **30** could be obtained selectively as well by an isomerization method [33–34]. From both acyclic precursors **29** and **30** the macrocycles **5** and **6** could be obtained analogously to **7**. In all cases and as expected, the new stilbene bridge was formed in the *Z* geometry exclusively. For the new compounds **5**–**7** conformational studies by measuring temperature dependent NMR spectra clearly indicated significant rotational barriers of about 70 kJ/mol for the biaryl axis ***B*** (see Supporting Information for each compound). These values, however, in comparison with the calculation for compound **3** (see Figure 3) indicate that the conformational flexibility of the macrocycles now synthesized is not significantly diminished with respect to this second biaryl axes. This result is also in accordance with the only moderately enhanced ring strain predicted for **5**–**7** compared to the parent compounds **3**/**4** (refer to Table 1).

It should be noted that the hydrogenation conditions did not lead to any reduction of the aldehyde groups but at an increased hydrogen pressure the saturated bibenzyl **31** was obtained which could be cyclized by the now improved procedure to the tetramethyl ether **3** of isoplagiochin C (**1**).

**Scheme 8:**
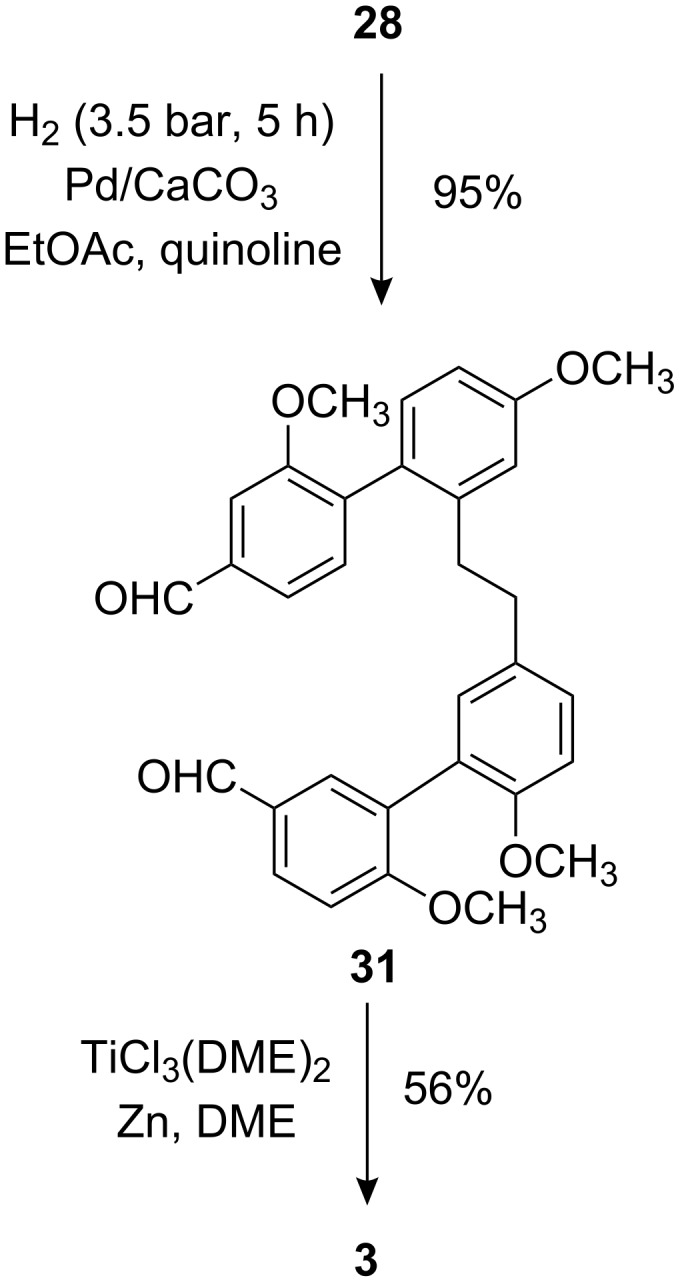
Synthesis of the known macrocycle **3** via McMurry reaction.

## Conclusion

By a new, flexible and straightforward pathway the macrocyclic framework of new isoplagiochin derivatives with enhanced ring strain was synthesized. A Sonogashira-type reaction allowed the flexible introduction of two-carbon aryl bridges. The McMurry protocol now should be considered as a powerful tool for the cyclization step in the synthesis of macrocycles of different bisbibenzyl types. The rotational barrier of the biaryl axis “***B***”, still highly flexible in the natural compounds **1** and **2**, is increased by simple modification, e. g. etherification. However, the flexibility and stability of the molecule is only slightly modified by introducing an unsaturated two-carbon bridge between rings b and c.

## Supporting Information

File 1 Full experimental details and characterization data.
