# Supplementary material for: Ring strain and total syntheses of modified macrocycles of the isoplagiochin type
Source: Beilstein J Org Chem. 2009 Dec 1;5:71. doi: 10.3762/bjoc.5.71 (PMC2839535; doi:10.3762/bjoc.5.71)
Supplement: File 1 — Full experimental details and characterization data. [file Beilstein_J_Org_Chem-05-71-s001.pdf]

Supporting Information for

Ring strain and total syntheses of modified macrocycles of the isoplagiochin type

Andreas Speicher*, Timo Backes, Kerstin Hesidens and Jürgen Kolz

Address: Department 8.1 Chemistry – Organic Chemistry, Saarland University, 66041 Saarbrücken, Germany

Email: Andreas Speicher - anspeich@mx.uni-saarland.de

* Corresponding author

Full experimental details and characterization data

General

The ^1H (500 MHz) and ^{13}C NMR (125 MHz) spectra were recorded on a Bruker DRX 500 spectrometer referencing to solvent or TMS with the chemical shift recorded as δ values in ppm. Coupling constants (*J*) are given in Hertz, and multiplicity is defined as follows: s = singlet, d = doublet, dd = doublet of doublet, t = triplet, q = quartet, m = multiplet, br = broad. The infrared (IR) spectra were recorded in terms of cm^{-1} on a Bruker Tensor 27 FT-IR spectrometer. Mass spectra were obtained on a Finnigan MAT 95. The melting points (mp) were determined on a Büchi melting point apparatus (Dr. Tottoli). Column chromatography was performed on a silica gel 60 (63–260 µm).

Temperature dependent ^1H NMR experiments for the tetramethylether 3 of isoplagiochin C and assignment of NMR data

The ^1H NMR spectrum of **3** was measured at different temperatures between −30 °C and +60 °C in $CDCl_3$. Coalescence of the signals for the four methoxy groups was observed at +15 °C (two sets), +40 °C and +55 °C (see Figure 3 in the main document).

The rotation barrier was approximated according to the formula

$$\Delta G^{\#} = 19.1 \cdot 10^{-3} \cdot T_C \cdot (9.97 + \log T_C - \log|\nu_A - \nu_B|)$$

(T_C: coalescence temperature in K,

ν_A, ν_B: resonance frequencies in Hz of the separated signals)

resulting in values of 66.0 to 66.4 kJ/mol.

For the exact assignment of NMR data especially for the four methoxy groups 2D NMR spectra (COSY, HSQC, HMBC) were measured at +100 °C in $[D_6]$-DMSO.

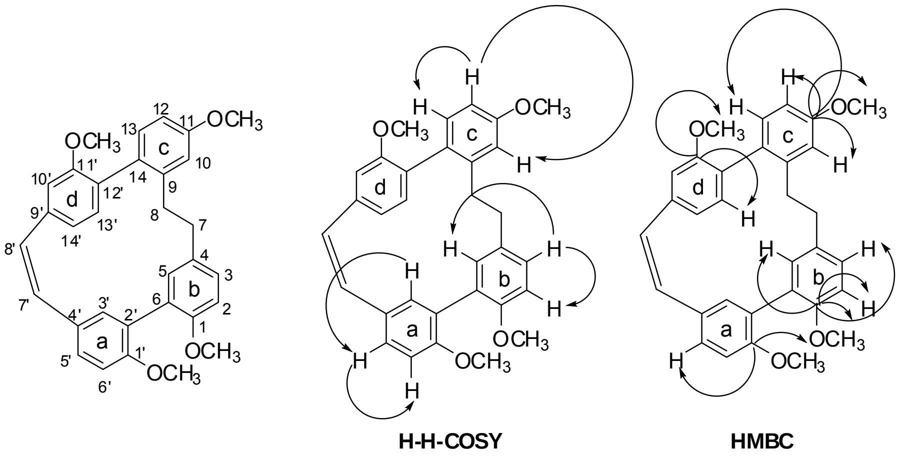

Figure 4: Typical NMR correlations for **3**.

Table 2: Assignment of 2D NMR data for compound **3** ([D$_6$]-DMSO, 100 °C).

Position	δ C (ppm)	δ H (ppm)	Position	δ C (ppm)	δ H (ppm)
1	154.8	–	1’	155.8	–
2	110.9	6.80 (d)	2’	128.2	
3	126.3	7.04 (d)	3’	132.3	6.89 (s_{br})
4	134.5	–	4’	128.0	–
5	132.2	6.40 (d)	5’	128.6	7.25 (dd)
6	127.6	–	6’	111.0	6.97 (d)
7	36.0	2.00–2.70 (m)	7’	129.3	6.65 (d)
8	37.0	2.00–2.70 (m)	8’	128.1	6.70 (d)
9	142.3	–	9’	138.9	–
10	113.5	6.92 (d)	10’	111.4	6.89 (s_{br})
11	158.3	–	11’	156.7	–
12	110.7	6.80 (d)	12’	not separated	–
13	130.2	7.04 (d)	13’	130.9	7.08 (d)
14	129.6	–	14’	120.0	6.84 (d)
**1-OCH$_3$**	**55.2**	**3.69 (s)**	**1’-OCH$_3$**	**54.9**	**3.75 (s)**
**11-OCH$_3$**	**54.6**	**3.82 (s)**	**11’-OCH$_3$**	**55.0**	**3.55 (s)**

2-(3-Bromo-4-methoxyphenyl)-1,3-dioxane (11)

A mixture of 3-bromo-4-methoxybenzaldehyde (**10**, 25.0 g, 116 mmol), triethyl orthoformate (19.0 g, 128 mmol), 1,3-propanediol (35.4 g, 464 mmol, 33.8 mL) and tetrabutylammonium tribromide (560 mg, 1.16 mmol) was stirred for 1 h at room

temperature. The reaction mixture was taken up in EtOAc (200 mL), washed with saturated $NaHCO_3$ (2 × 100 mL) and H_2O (3 × 100 mL), dried ($MgSO_4$) and concentrated yielding a colorless oil which solidifies on standing, 31.1 g (98%), mp 67 °C.

IR: ν (cm^{-1}) = 2955, 2840, 1620, 1510, 1460, 1370, 1295, 1235, 1205, 1180, 1130, 1150, 1050, 1020, 980, 950, 880, 850, 810, 780, 670.

^{1}H NMR ($CDCl_3$): δ (ppm) = 7.69 (d, *J* = 1.9 Hz, 1 H, Ar-H), 7.38 (dd, J_1 = 8.5 Hz, J_2 = 1.9 Hz, 1 H, Ar-H), 6.87 (d, *J* = 8.5 Hz, 1 H, Ar-H), 5.43 (s, 1 H, –OCHO–), 4.26–4.21 (combined signals, 2 H, $–OCH_2–$), 4.00–3.92 (combined signals, 2 H, $–OCH_2–$), 3.88 (s, 3 H, $–OCH_3$), 2.25–2.13 (m,1 H, HCH), 1.41 (d, *J* = 13.7 Hz, 1 H, HCH).

^{13}C NMR ($CDCl_3$): δ (ppm) = 156.16, 132.69, 131.25, 126.29, 111.46, 100.53 (–OCHO–), 67.36, 56.31, 25.70.

5-Formyl-2-methoxyphenylboronic acid (12)

To the bromoarene **11** (10.5 g, 38.4 mmol) in anhydrous THF (200 mL) was added dropwise *n*-BuLi in *n*-hexane (2.5 M, 16.8 mL, 42.0 mmol) at T < −70 °C. Stirring was continued for 4 h at *T* < −70 °C. Trimethyl borate (4.98 g, 47.9 mmol) was added, the mixture was allowed to warm up to room temperature overnight and hydrolyzed with 2 M HCl (100 mL). The aqueous layer was extracted with Et_2O (2 × 100 mL) and the combined organic layers were extracted with 2 M NaOH (2 × 100 mL). The alkaline solution was acidified to pH 1 (conc. HCl). The boronic acid as precipitate was filtered off and dried *in vacuo* ($CaCl_2$); pale yellow solid, 5.39 g (78%), mp 160 °C.

IR: ν (cm^{-1}) = 3235 (OH), 1690 (C=O), 1660, 1600, 1575, 1495, 1465, 1425, 1400, 1380, 1250, 1210, 1180, 1140, 1045, 1015, 895, 840, 770, 755, 720, 665.

^{1}H NMR ([D_6]-DMSO): δ (ppm) = 9.87 (s, 1 H, CHO), 8.04 (d, J = 2.1 Hz, 1 H, Ar-H), 7.92 (dd, J_1 = 8.5 Hz, J_2 = 2.2 Hz, 1 H, Ar-H), 7.92 (s, 2 H, −$B(OH)_2$), 7.15 (d, J = 8.5 Hz, 1 H, Ar-H), 3.88 (s, 3 H, −OCH_3).

^{13}C NMR ([D_6]-DMSO): δ (ppm) = 191.57 (−CHO), 167.85, 136.86, 133.65, 129.10, 110.76, 55.82.

5'-(1,3-Dioxan-2-yl)-6,2'-dimethoxybiphenyl-3-carbaldehyde (13)

The bromoarene **11** (6.64 g, 24.3 mmol) and the boronic acid **12** (4.83 g, 26.8 mmol) were dissolved in a mixture of toluene (45 mL), 2 M Na_2CO_3 (22 mL) and EtOH (30 mL) and degassed with argon. Tetrakis(triphenylphosphane)-palladium(0) (867 mg, 0.75 mmol) was added and the mixture was heated to reflux for 24 h, cooled to room temperature and filtered through an alumina pad eluting with Et_2O. H_2O (200 mL) was added and the mixture was extracted with Et_2O (4 × 200 mL). The combined organic layers were dried ($MgSO_4$) and concentrated. The crude product was purified filtrating through an alumina pad (basic, activity III) in CH_2Cl_2; pale yellow oil which solidifies; 7.10 g (89%), mp 105 °C.

IR: ν (cm^{-1}) = 2965, 2840, 2725, 1690 (C=O), 1605, 1505, 1465, 1445, 1380, 1275, 1210, 1175, 1150, 1130, 1105, 1050, 1000, 940, 900, 870, 855, 820, 790, 735, 705.

^{1}H NMR ($CDCl_3$): δ (ppm) = 9.90 (s, 1 H, −CHO), 7.88 (dd, J_1 = 8.5 Hz, J_2 = 2.2 Hz, 1 H, Ar-H), 7.77 (d, J = 2.2 Hz, 1 H, Ar-H), 7.47 (dd, J_1 = 8.5 Hz, J_2 = 2.2 Hz, 1 H, Ar-H), 7.34 (d, J = 2.2 Hz, 1 H, Ar-H), 7.04 (d, J = 8.5 Hz, 1 H, Ar-H), 6.95 (d, J = 8.5 Hz, 1 H, Ar-H), 5.49 (s, 1 H, –OCHO–), 4.27–4.24 (combined signals, 2 H, −OCH_2−), 4.00–3.95 (combined signals, 2 H, −OCH_2−), 3.83 (s, 3 H, −OCH_3), 3.76 (s, 3 H, −OCH_3), 2.26–2.16 (m,1 H, HCH), 1.43 (d, J = 13.6 Hz, 1 H, HCH).

^{13}C NMR ($CDCl_3$): δ (ppm) = 190.99 (–CHO), 162.27, 157.45, 133.54, 131.16, 131.12, 129.55, 129.08, 128.67, 126.98, 126.36, 110.77, 110.72, 101.45 (–OCHO–), 67.38, 55.90, 55.85, 25.78.

2-(5'-Ethynyl-6,2'-dimethoxybiphenyl-3-yl)-1,3-dioxane (14)

Bromomethyltriphenylphosphonium bromide (11.6 g, 26.8 mmol) was dissolved in anhydrous THF (200 mL) under argon atmosphere and cooled to −78 °C. *t*-BuOK (31.6 g, 56.3 mmol, 20% w/w in anhydrous THF) was added slowly and the mixture was stirred for 1 h at −78 °C. To the orange solution the aldehyde **13** (7.32 g, 22.3 mmol) in anhydrous THF (200 mL) was added dropwise at T < −70 °C. The mixture was allowed to warm to room temperature within 6 h and taken up in Et_2O (300 mL). The organic layer was washed with H_2O (3 × 200 mL), brine (200 mL), dried ($MgSO_4$) and concentrated. The crude product was purified by CC (basic alumina, activity III, CH_2Cl_2), pale yellow solid, 6.73 g (93%), mp 105 °C.

IR: ν (cm^{-1}) = 3272 (C≡C–H), 2965, 2838, 2210, 1760, 1600, 1583, 1492, 1463, 1440, 1367, 1331, 1271, 1234, 1179, 1147, 1131, 1116, 1100, 1045, 1014, 967, 952, 894, 876, 864, 809, 624, 614.

^{1}H NMR ($CDCl_3$): δ (ppm) = 7.45 (dd, J_1 = 8.5 Hz, J_2 = 2.2 Hz, 1 H, Ar-H), 7.44 (dd, J_1 = 8.5 Hz, J_2 = 2.2 Hz, 1 H, Ar-H), 7.37 (d, J = 2.2 Hz, 1 H, Ar-H), 7.31 (d, J = 2.2 Hz, 1 H, Ar-H), 6.93 (d, J = 8.5 Hz, 1 H, Ar-H), 6.86 (d, J = 8.5 Hz, 1 H, Ar-H), 5.48 (s, 1 H, –OCHO–), 4.27–4.22 (combined signals, 2 H, –OCH_2–), 3.99–3.94 (combined signals, 2 H, –OCH_2–), 3.75 (s, 3 H, –OCH_3), 3.74 (s, 3 H, –OCH_3), 2.97 (s, 1 H, –C≡CH), 2.25–2.15 (m,1 H, HCH), 1.42 (m, 1 H, HCH).

^{13}C NMR ($CDCl_3$): δ (ppm) = 157.69, 157.48, 135.24, 132.75, 131.04, 129.18, 128.18, 126.77, 126.65, 113.84, 110.77, 110.75, 101.52 (–OCHO–), 83.83 (–$\underline{C}$≡C–), 75.61 (–C≡$\underline{C}$–), 67.36, 55.88, 55.69, 25.79.

MS (CI, 120 eV): *m/z* (%) = 324 (100, M^+), 323 (40), 293 (10), 266 (20), 248 (5), 208 (5), 193 (10), 165 (3), 87 (18).

5'-[(2-Bromo-5-methoxyphenyl)ethynyl]-6,2'-dimethoxybiphenyl-3-carbaldehyde (16)

To 4-Bromo-3-iodoanisole (**15**, 4.46 g, 14.3 mmol), CuI (273 mg, 1.43 mmol), *N,N*-dimethylglycine hydrochloride (596 mg, 4.28 mmol) and K_2CO_3 (5.91 g, 42.8 mmol) in a pre-conditioned oxygen free argon atmosphere was added the alkyne **14** (5.56 g (17.1 mmol) in DMF (40 mL) and H_2O (1 mL, both degassed). The mixture was heated to 100 °C for 12 h (argon atmosphere), cooled and taken up in EtOAc (200 mL) and H_2O (100 mL). The layers were separated and the aqueous layer was extracted with EtOAc (2 × 50 mL). The combined organic layers were washed with H_2O (2 × 50 mL), dried ($MgSO_4$) and concentrated. The residue was taken up in 1,4-dioxane (50 mL) and hydrolyzed with 2 M HCl (20 mL) for 1 h at room temperature. The mixture was extracted with EtOAc (3 × 50 mL), washed (3 × 100 mL H_2O), dried ($MgSO_4$) and concentrated. The crude product was purified by CC (silica gel, CH_2Cl_2); colorless solid, 4.58 g (71%), mp 100–103 °C.

IR: ν (cm^{-1}) = 2935, 2837, 2205 (C≡C), 1684 (C=O), 1584, 1491, 1460, 1439, 1391, 1249, 1172, 1144, 1113, 1040, 1016, 959, 905, 852, 813.

^{1}H NMR ($CDCl_3$): δ (ppm) = 9.92 (s, 1 H, –CHO), 7.90 (dd, J_1 = 8.5 Hz, J_2 = 2.2 Hz, 1 H, Ar-H), 7.79 (d, J = 2.2 Hz, 1 H, Ar-H), 7.59 (dd, J_1 = 8.5 Hz, J_2 = 2.2 Hz, 1 H, Ar-H), 7.46 (combined signals, 2 H, Ar-H), 7.08 (d, J = 8.5 Hz, 1 H, Ar-H), 7.05 (d, J =

2.8 Hz, 1 H, Ar-H), 6.96 (d, J = 8.5 Hz, 1 H, Ar-H), 6.74 (dd, J_1 = 8.8 Hz, J_2 = 2.8 Hz, 1 H, Ar-H), 3.87 (s, 3 H, $–OCH_3$), 3.80 (s, 3 H, $–OCH_3$), 3.79 (s, 3 H, $–OCH_3$).

^{13}C NMR ($CDCl_3$): δ (ppm) = 190.84 (–CHO), 162.15, 158.51, 157.54, 134.69, 133.31, 133.02, 132.95, 131.56, 129.65, 127.71, 126.82, 126.24, 117.62, 116.25, 116.21, 114.92, 111.07, 110.93, 93.69 (–C≡C–), 87.15 (–C≡C–), 56.01, 55.82, 55.57.

MS (CI, 120 eV): *m/z* (%) = 452 (40, M^+), 450 (35), 373 (20), 372 (100), 344 (20), 328 (5), 279 (2).

by-product: dialkyne **17**, mp 145–150 °C

^{1}H NMR ($CDCl_3$): δ (ppm) = 9.90 (s, 1 H, –CHO), 7.89 (dd, J_1 = 8.5 Hz, J_2 = 2.2 Hz, 1 H, Ar-H, 7.75 (d, J = 2.2 Hz, 1 H, Ar-H), 7.53 (dd, J_1 = 8.8 Hz, J_2 = 2.2 Hz, 1 H, Ar-H), 7.40 (d, J = 2.2 Hz, 1 H, Ar-H), 7.07 (d, J = 8.5 Hz, 1 H, Ar-H), 6.92(d, J = 8.8 Hz, 1 H, Ar-H), 3.86 (s, 3 H, $–OCH_3$), 3.78 (s, 3 H, $–OCH_3$).

^{13}C NMR ($CDCl_3$): δ (ppm) = 190.78 (–CHO), 162.04, 157.78, 135.46, 133.79, 133.31, 131.57, 129.62, 127.34, 126.80, 113.87, 111.13, 110.97, 81.13 (Ar–C≡C–C≡C), 73.24 (Ar–C≡C–C≡C), 56.00, 55.79.

4-Iodo-3-methoxybenzoic acid (19)

step (1): 4-Iodo-3-hydroxybenzoic acid

3-Hydroxybenzoic acid (**18**, 20.8 g, 150 mmol) was dissolved in 2 M NaOH (200 mL) and a solution of iodine (35.1 g, 138 mmol) and KI (27.5 g, 165 mmol) in H_2O (150 mL) was added dropwise. Stirring was continued for 20 min, concentrated HCl (180 mL) was added and the precipitate was filtered off, re-crystallized from EtOH–H_2O (1:5) and dried *in vacuo* (P_4O_{10}); colorless solid, 25.3 g (64%), mp 219–221 °C.

step (2): 4-Iodo-3-methoxybenzoic acid methyl ester

4-Iodo-3-hydroxybenzoic acid (10.0 g, 37.9 mmol), dimethyl sulfate (21.8 g, 189 mmol, 18.0 mL) and K_2CO_3 (15.7 g, 114 mmol) in acetone (100 mL) were heated to reflux for 12 h. H_2O (200 mL) was added, the mixture was stirred for 12 h at room temperature and extracted with EtOAc (2 × 100 mL). The combined organic layers were dried ($MgSO_4$) and concentrated. The residue was filtered through a silica gel pad ($CHCl_3$); colorless oil which solidifies; 10.7 g (97%), mp 48–49 °C.

step(3): 4-Iodo-3-methoxybenzoic acid (**19**)

4-Iodo-3-methoxybenzoic acid methyl ester (4.60 g, 15.8 mmol) was dissolved in 2 M NaOH (100 mL) and stirred for 24 h at room temperature. The alkaline solution was acidified with concentrated HCl and the precipitate was filtered off, washed (H_2O) and dried *in vacuo* over P_4O_{10}; colorless solid, 4.08 g (93%), mp 219–220 °C.

IR: ν (cm^{-1}) = 2967 (OH), 2840, 2654, 2533, 1689, 1572, 1463, 1421, 1296, 1250, 1188, 1121, 1041, 1016, 933, 879, 828, 761, 601.

^{1}H NMR ($CDCl_3$): δ (ppm) = 7.91 (d, J = 8.0 Hz, 1 H, Ar-H), 7.44 (d, J = 1.8 Hz, 1 H, Ar-H), 7.32 (dd, J_1 = 8.0 Hz, J_2 = 1.8 Hz, 1 H, Ar-H), 3.91 (s, 3 H, $–OCH_3$).

^{13}C NMR ($CDCl_3$): δ (ppm) = 166.77 (–COOH), 157.83, 139.24, 132.57, 123.14, 111.25, 92.49 (C_{Ar}–I), 56.45.

MS (CI, 120 eV): *m/z* (%) = 278 (50, M^+), 263 (68), 261–257 (45–100), 201 (35), 179 (35), 152 (18), 121 (10), 87 (40).

(4-Iodo-3-methoxyphenyl)methanol (20)

To thionyl chloride (50 mL, 0.69 mol) was added the benzoic acid **19** (14.5 g, 52.0 mmol) and the solution was heated to reflux for 1 h. The excess of thionyl chloride was distilled off, the crude benzoyl chloride was dissolved in anhydrous 1,4-dioxane

(100 mL), $NaBH_4$ (5.93 g, 156 mmol) was added and the mixture was heated to 100 °C for 1 h. H_2O (10 mL) was added, most of the solvent was removed *in vacuo* and the residue was extracted with CH_2Cl_2 (3 × 50 mL). The combined organic layers were dried ($MgSO_4$ and concentrated. Purification by CC (short pad of silica gel, $CHCl_3$) yielded a colorless oil; 10.6 g (77%).

IR: ν (cm^{-1}) = 3219 (OH), 3000, 2934, 2831, 1686, 1588, 1571, 1501, 1476, 1459, 1399, 1256, 1186, 1163, 1127, 1034, 1012, 868, 847, 803, 759.

^{1}H NMR ($CDCl_3$): δ (ppm) = 7.72 (d, *J* = 7.9 Hz, 1 H, Ar-H), 6.86 (d, *J* = 1.6 Hz, 1 H, Ar-H), 6.69 (dd, J_1 = 7.9 Hz, J_2 = 1.6 Hz, 1 H, Ar-H), 4.65 (s, 2 H, Ar–CH_2–O), 3.89 (s, 3 H, –OCH_3).

^{13}C NMR ($CDCl_3$): δ (ppm) = 158.29, 142.94, 139.39, 120.74, 109.44, 84.59 (C_{Ar}–I), 64.76 (Ar–<u>C</u>H_2–O), 56.32.

MS (CI, 120 eV): *m/z* (%) = 264 (100, M^+), 247 (10), 137 (3), 109 (8).

2-(4-Iodo-3-methoxybenzyloxy)tetrahydropyran (21)

The iodobenzyl alcohol **20** (2.52 g, 9.39 mmol) was dissolved in anhydrous CH_2Cl_2 (25 mL), 3,4-dihydro-*2H*-pyran (1.98 g, 2.12 mL, 23.5 mmol) and *p*-toluenesulfonic acid monohydrate (36.3 mg, 0.19 mmol) were added and the mixture was stirred for 16 h at room temperature. The solvent was removed and the residue was purified by CC (silica gel, CH_2Cl_2); colorless oil, 2.50 g (76%).

IR: ν (cm^{-1}) = 2937, 2865, 1574, 1461, 1405, 1347, 1279, 1256, 1200, 1169, 1118, 1075, 1032, 1013, 974, 903, 869, 811.

^{1}H NMR ($CDCl_3$): δ (ppm) = 7.72 (d, *J* = 7.9 Hz, 1 H, Ar-H), 6.85 (d, *J* = 1.9 Hz, 1 H, Ar-H), 6.72 (dd, J_1 = 7.9 Hz, J_2 = 1.9 Hz, 1 H, Ar-H), 4.74, 4.47 (2 d, *J* = 12.3 Hz, 2 H,

Ar–CH_2–O), 4.69 (t, *J* = 3.5 Hz, 1 H, O–CH–O), 3.93–3.89 (m, 1 H, –CH_2–O), 3.89 (s, 3 H, –OCH_3), 3.57–3.52 (m, 1 H, –CH_2–O), 1.87–1.50 (m, 6 H, –CH_2–).

^{13}C NMR ($CDCl_3$): δ (ppm) = 158.15, 140.24, 139.26, 121.85, 110.39, 97.83 (O–<u>C</u>H–O), 84.59 (C_{Ar}–I), 68.29, 62.31, 56.30, 30.57, 25.44, 19.41.

MS (CI, 120 eV): *m/z* (%) = 391 (100), 348 (30, M^+), 329 (4), 267 (2), 253 (15), 251 (2), 183 (2), 169 (45), 168 (8), 167 (22), 166 (5), 85 (2).

{2-Methoxy-4-[(tetrahydropyran-2-yloxy)methyl]phenyl}boronic acid (22)

A solution of the iodoarene **21** (8.95 g, 25.7 mmol) in anhydrous THF (200 mL) was cooled to −78 °C, *n*-butyllithium in *n*-hexane (2.5 M, 12.5 mL, 31.1 mmol) was added dropwise at $T < -70$ °C and stirring was continued for 30 min. Trimethyl borate (8.06 g, 8.58 mL, 77.3 mmol) was added at $T < -70$ °C and the mixture was stirred for 1 h and then allowed to warm to room temperature. H_2O (200 mL) was added and the mixture was extracted with Et_2O (3 × 150 mL). From the organic layer the boronic acid was extracted with 2 M NaOH (3 × 100 mL) and the alkaline solution was slightly acidified with diluted HCl to pH 6–7. The boronic acid was re-extracted with Et_2O (3 × 150 mL), dried ($MgSO_4$) and concentrated; colorless oil, 4.20 g (61%).

IR: ν (cm^{-1}) = 3394 (OH), 2940, 2869, 1611, 1566, 1500, 1454, 1416, 1322, 1246, 1162, 1118, 1032, 976, 948, 904, 867, 814, 744.

^{1}H NMR ($CDCl_3$): δ (ppm) = 7.81(d, *J* = 7.6 Hz, 1 H, Ar-H), 7.02 (dd, not resolved, J_1 = 7.6 Hz, 1 H, Ar-H), 6.95 (d, not resolved, 1 H, Ar-H), 5.91 (s, 2 H, –$B(OH)_2$), 4.81, 4.54 (2 d, *J* = 12.6 Hz, 2 H, Ar–CH_2–O), 4.71 (t, *J* = 3.5 Hz, 1 H, O–CH–O), 3.93 (s, 3 H, –OCH_3), 3.75 (m, 1 H, –CH_2–O), 3.56 (m, 1 H, –CH_2–O), 1.87–1.53 (m, 6 H, –CH_2–).

^{13}C NMR ($CDCl_3$): δ (ppm) = 164.76, 143.72, 136.87, 120.36, 109.14, 97.92 (O–C̲H–O), 68.62, 62.30, 55.51, 30.59, 25.46, 19.42.

MS (CI, 120 eV): *m/z* (%) = 238 (80), 221 (100), 169 (60), 137 (25), 85 (27).

Vanillin triflate (24)

To a solution of vanillin (**23**) (3.40 g, 22.3 mmol) and pyridine (4.21 g, 53.4 mmol) in anhydrous CH_2Cl_2 (150 mL) was added dropwise trifluoromethanesulfonic anhydride (12.6 g, 44.6 mmol) at 0 °C. The mixture was allowed to warm to room temperature and stirred for additional 5 h. Saturated $NaHCO_3$ (200 mL) and H_2O (200 mL) were added and the mixture was extracted with EtOAc (3 × 150 mL). The organic layer was dried ($MgSO_4$) and concentrated. Purification by CC (silica gel pad, CH_2Cl_2) yielded a colorless oil, 5.70 g (90%).

IR: ν (cm^{-1}) = 2984, 2853, 1725, 1692 (C=O), 1605, 1499, 1462, 1422, 1323, 1290, 1248, 1197, 1156, 1135, 1102, 1031, 923, 865, 837, 770, 754, 737, 713, 644, 613.

^{1}H NMR ($CDCl_3$): δ (ppm) = 9.99 (s, 1 H, –CHO), 7.57 (d, *J* = 1.9 Hz, 1 H, Ar-H), 7.52 (dd, J_1 = 8.2 Hz, J_2 = 1.9 Hz, 1 H, Ar-H), 7.42 (d, *J* = 8.2 Hz, 1 H, Ar-H), 4.00 (s, 3 H, –OCH_3).

^{13}C NMR ($CDCl_3$): δ (ppm) = 190.33 (–CHO), 152.29, 142.78, 136.85, 124.11, 123.24, 118.74 (q, $J_{C–F}$ = 312 Hz), 111.84, 56.54.

MS (CI, 120 eV): *m/z* (%) = 84 (M^+, 55), 238 (15), 179 (15), 151 (100), 137 (8), 95 (30), 79 (8).

Trifluoromethanesulfonic acid 4-(1,3-dioxan-2-yl)-2-methoxyphenyl ester (25)

A mixture of vanillin triflate (**24**) (5.68 g, 20.0 mmol), triethyl orthoformate (3.27 g, 22.0 mmol, 3.65 mL), 1,3-propanediol (6.15 g, 80.0 mmol, 5.88 mL) and tetrabutylammonium tribromide (96.7 mg, 1.16 mmol) was stirred for 1 h at room temperature. The reaction mixture was taken up in EtOAc (100 mL), washed with saturated $NaHCO_3$ (2 × 50 mL) and H_2O (3 × 50 mL), dried ($MgSO_4$) and concentrated; colorless oil, 6.79 g (99%).

IR: ν (cm^{-1}) = 2858, 1725, 1610, 1503, 1466, 1417, 1378, 1311, 1286, 1247, 1202, 1138, 1100, 1023, 999, 882, 865, 821, 782.

^{1}H NMR ($CDCl_3$): δ (ppm) = 7.22 (d, J = 1.9 Hz, 1 H, Ar-H), 7.20 (d, J = 8.2 Hz, 1 H, Ar-H), 7.08 (dd, J_1 = 8.2 Hz , J_2 = 1.9 Hz, 1 H, Ar-H), 5.48 (s, 1 H, –OCHO–), 4.30–4.25 (combined signals, 2 H, $–OCH_2–$), 4.03–3.95 (combined signals, 2 H, $–OCH_2–$), 3.93 (s, 3 H, $–OCH_3$), 2.28–2.16 (m,1 H, HCH), 1.49–1.44 (m, 1 H, HCH).

^{13}C NMR ($CDCl_3$): δ (ppm) = 151.31, 140.14, 138.84, 122.17, 118.80 (q, $J_{C–F}$ = 321 Hz), 118.72, 111.02, 100.39 (–OCHO–), 67.43, 56.23, 25.67.

2-[3-Methoxy-4-(4,4,5,5-tetramethyl-1,3,2-dioxaborolan-2-yl)phenyl]-1,3-dioxane (26)

To a solution of $PdCl_2$(dppf) (487 mg, 0.60 mmol) in anhydrous 1,4-dioxane (120 mL) was added the triflate **25** (6.78 g, 19.8 mmol), triethylamine (8.34 mL, 59.5 mmol) and pinacolborane (2.53 g, 19.8 mmol) and the mixture was heated to 100 °C for 6 h. Additional pinacolborane (2.53 g, 19.8 mmol) was added and heating was continued

for 6 h. After cooling, the black reaction mixture was taken up in benzene (200 mL), washed with H_2O (3 × 100 mL) and brine (100 mL), dried ($MgSO_4$) and concentrated. Purification by CC (basic alumina, EtOAc/*n*-hexane 1:1) yielded a colorless oil, 3.46 g (55%).

IR: ν (cm^{-1}) = 2974, 2853, 1724, 1614, 1568, 1507, 1458, 1420, 1372, 1342, 1316, 1278, 1253, 1237, 1215, 1194, 1169, 1144, 1100, 1066, 1037, 996, 963, 951, 915, 894, 878, 856, 821, 786, 696.

^{1}H NMR ($CDCl_3$): δ (ppm) = 7.65 (d, *J* = 7.6 Hz, 1 H, Ar-H), 7.02 (combined signals, 2 H, Ar-H), 5.48 (s, 1 H, –OCHO–), 4.30–4.25 (combined signals, 2 H, –OCH_2–), 4.02–3.93 (combined signals, 2 H, –OCH_2–), 3.85 (s, 3 H, –OCH_3), 2.24 (m, 1 H, –HCH–), 1.45 (d, *J* = 13.6 Hz, 1 H, HCH), 1.34 (s, 12 H, –CH_3).

^{13}C NMR ($CDCl_3$): δ (ppm) = 164.37, 143.03, 136.74, 117.95, 108.01, 101.46 (–OCHO–), 83.43 (–O$\underline{C}$(CH_3)$_2$), 67.40, 55.91, 25.79, 24.88, 24.83 (–OC($\underline{C}H_3$)$_2$).

MS (CI, 120 eV): *m/z* (%) = 320 (M^+, 100), 319 (55), 291 (22), 277 (5), 219 (6), 211 (35), 193 (45), 163 (8), 137 (2), 101 (4), 87 (17).

5'-(4'-Hydroxymethyl-4,2'-dimethoxybiphenyl-2-ylethynyl)-6,2'-dimethoxybiphenyl-3-carbaldehyde (27)

The bromoalkyne **16** (1.10 g, 2.44 mmol) and the boronic acid **22** (0.97 g, 3.65 mmol) were dissolved in a mixture of toluene (165 mL), 1 M Na_2CO_3 (35 mL) and EtOH (65 mL) and degassed with argon. Tetrakis(triphenylphosphane)-palladium(0) (138 mg, 118 µmol) was added and the mixture was heated to reflux for 12 h, cooled to room temperature and filtered through a silica gel pad eluting with Et_2O. H_2O (65 mL) was added and the mixture was extracted with Et_2O (3 × 80 mL). The combined organic layers were dried ($MgSO_4$) and concentrated. The crude product was purified

filtrating through an alumina pad (basic, activity **III**) in CH_2Cl_2; pale yellow oil which solidifies; 7.10 g (89%), mp 105 °C. The residue was taken up in 1,4-dioxane (60 mL) and hydrolyzed with 2 M HCl (35 mL) for 1 h at room temperature. The mixture was extracted with EtOAc (3 × 50 mL), washed (3 × 100 mL H_2O), dried ($MgSO_4$) and concentrated. The crude product was purified by CC (silica gel, EtOAc/*n*-hexane 1:1); pale yellow solid, 700 mg (56%), mp 67–70 °C

IR: ν (cm^{-1}) = 3359 (OH), 3001, 2933, 2836, 2207 (C≡C), 2039, 1982, 1899, 1732, 1686 (C=O), 1595, 1575, 1490, 1460, 1409, 1318, 1249, 1169, 1146, 1123, 1018, 901, 855, 811, 771.

^{1}H NMR ($CDCl_3$): δ (ppm) = 9.92 (s, 1 H, –CHO), 7.89 (dd, J_1 = 8.5 Hz, J_2 = 2.2 Hz, 1 H, Ar-H), 7.72 (d, *J* = 2.2 Hz, 1 H, Ar-H), 7.33 (d, *J* = 7.6 Hz, 1 H, Ar-H), 7.28 (d, *J* = 8.5 Hz, 1 H, Ar-H), 7.25 (dd, J_1 = 8.5 Hz, J_2 = 2.2 Hz, 1 H, Ar-H), 7.12 (d, *J* = 2.5 Hz, 1 H, Ar-H), 7.08 (combined signals, 2 H, Ar-H), 7.00 (d, not resolved, 1 H, Ar-H), 6.96 (dd, not resolved, 1 H, Ar-H), 6.92 (dd, J_1 = 8.5 Hz, J_2 = 2.5 Hz, 1 H, Ar-H), 6.86 (d, *J* = 8.5 Hz, 1 H, Ar-H), 4.66 (s, 2 H, Ar–C$\underline{H}_2$–OH), 3.85 (s, 3 H, –OCH_3), 3.85 (s, 3 H, –OCH_3), 3.79 (s, 3 H, –OCH_3), 3.75 (s, 3 H, –OCH_3).

^{13}C NMR ($CDCl_3$): δ (ppm) = 191.02 (–CHO), 171.16, 162.14, 158.36, 157.33, 156.94, 141.80, 134.48, 134.52, 133.43, 132.76, 132.39, 131.99, 131.83, 131.31, 129.60, 128.94, 127.91, 126.51, 124.21, 118.34, 116.14, 115.62, 114.53, 110.88, 109.40, 91.45 (–$\underline{C}$≡C–), 88.41 (–C≡$\underline{C}$–), 65.31 (Ar–$\underline{C}H_2$–OH), 56.00, 55.74, 55.69, 55.39.

MS (CI, 120 eV): *m/z* (%) = 508 (5, M^+), 404 (5), 279 (35), 277 (25), 274 (60), 257 (25), 214 (10), 163 (100), 154 (90), 137 (75), 85 (40).

5'-(4,2'-Dimethoxy-4'-formylbiphenyl-2-ylethynyl)-6,2'-dimethoxybiphenyl-3-carbaldehyde (28)

(a) by oxidation of **27**:

To a suspension of PCC on alumina (638 mg, 638 µmol, 1 mmol/g) in anhydrous CH_2Cl_2 (30 mL) was added the benzyl alcohol **27** (216 mg, 0.43 mmol) in CH_2Cl_2 (20 mL) with vigorous stirring for 24 h at room temperature. The slurry was filtered off rewashing with CH_2Cl_2 (20 mL) and concentrated. Purification by CC (silica gel, EtOAc/*n*-hexane (1:1) yielded a colorless solid, 183 mg (85%).

(b) by Suzuki reaction of **16** und **26**:

The bromoalkyne **16** (568 mg, 1.26 mmol) and the boronic ester **26** (1.00 g, 3.12 mmol) were dissolved in toluene (15 mL) an EtOH (3 mL) and degassed with argon. Na_2CO_3 (1.20 g, 11.4 mmol) in degassed H_2O (6 mL) and $Pd(PPh_3)_4$ (72.2 mg, 63.0 µmol) were added and the mixture was heated to reflux for 12 h. After cooling to room temperature H_2O (20 mL) was added and the mixture was extracted with Et_2O (3 × 25 mL). The combined organic layers were dried ($MgSO_4$) and concentrated. The residue was taken up in 1,4-dioxane (20 mL) and hydrolyzed with 2 M HCl (10 mL) for 1 h at room temperature. The mixture was extracted with EtOAc (3 × 15 mL), washed (3 × 30 mL H_2O), dried ($MgSO_4$) and concentrated. The crude product was purified by CC (silica gel, EtOAc/*n*-hexane 1:1); colorless solid, 469 mg (73%), mp 76–78 °C; HPLC: Nucleosil 100-5, 4.0 × 250 mm, EtOAc/*n*-hexane 30:70.

IR: ν (cm^{-1}) = 2938, 2836, 2204 (C≡C), 1685 (C=O), 1594, 1574, 1488, 1459, 1416, 1385, 1249, 1170, 1148, 1123, 1099, 1020, 1000, 958, 813.

^{1}H NMR ($CDCl_3$): δ (ppm) = 9.96 (s, 1 H, –CHO), 9.91 (s, 1 H, –CHO), 7.88 (dd, J_1 = 8.5 Hz, J_2 = 2.2 Hz, 1 H, Ar-H), 7.71 (d, J = 2.2 Hz, 1 H, Ar-H), 7.56 (d, J = 7.6 Hz, 1 H, Ar-H), 7.50 (dd, J_1 = 7.6 Hz, J_2 = 1.6 Hz, 1 H, Ar-H), 7.48 (d, J = 1.6 Hz, 1 H, Ar-

H), 7.29 (d, J = 8.5 Hz, 1 H, Ar-H), 7.18 (dd, J_1 = 8.5 Hz, J_2 = 2.2 Hz, 1 H, Ar-H), 7.13 (d, J = 2.8 Hz, 1 H, Ar-H), 7.09 (d, J = 2.2 Hz, 1 H, Ar-H), 7.06 (d, J = 8.5 Hz, 1 H, Ar-H), 6.94 (dd, J_1 = 8.5 Hz, J_2 = 2.8 Hz, 1 H, Ar-H), 6.85 (d, J = 8.5 Hz, 1 H, Ar-H), 3.86 (s, 3 H, –OCH_3), 3.85 (s, 3 H, –OCH_3), 3.84 (s, 3 H, –OCH_3), 3.75 (s, 3 H, –OCH_3).

^{13}C NMR ($CDCl_3$): δ (ppm) = 191.93, 190.84 (2-CHO), 162.03, 158.95, 157.87, 157.12, 136.94, 136.58, 134.41, 133.01, 132.45, 132.37, 132.25, 131.73, 131.00, 129.64, 127.62, 126.64, 124.16, 123.73, 116.41, 115.21, 114.58, 111.04, 110.96, 109.33, 92.02 (–<u>C</u>≡C–), 87.80 (–C≡<u>C</u>–), 55.99, 55.88, 55.76, 55.43.

MS (CI, 120 eV): *m/z* (%) = 507 (7, M^+), 506 (9), 405 (6), 377 (75), 376 (100), 285 (8), 272 (60), 270 (80), 202 (8), 137 (5), 89 (8), 88 (18).

(Z)-5'-[2-(4'-Formyl-4,2'-dimethoxybiphenyl-2-yl)-vinyl]-6,2'-dimethoxybiphenyl-3-carbaldehyde (29)

The alkyne **28** (200 mg, 0.40 mmol) was dissolved in EtOAc (50 mL) and Lindlar catalyst (5% Pd on $CaCO_3$, poisoned with $Pb(OAc)_2$; 168 mg, 78.7 µmol) as well as two drops of quinoline were added. The Parr hydrogenation apparatus was flushed several times with hydrogen and hydrogenation was performed for 2 h at 1.5 bar (20 psi). The catalyst was filtered off and the solvent removed *in vacuo*. The crude product was purified by CC (silica gel, EtOAc/*n*-hexane 2:1) yielding a pale yellow oil which solidifies on standing; 191 mg (95%), mp 50 °C; HPLC: Nucleosil 100-5, 4.0 × 250 mm, EtOAc/*n*-hexane 30:70.

IR: ν (cm^{-1}) = 3002, 2936, 2834, 2726, 1684 (C=O), 1595, 1573, 1499, 1460, 1414, 1384, 1264, 1246, 1229, 1194, 1178, 1165, 1150, 1120, 1021, 1000, 958, 809, 735.

^{1}H NMR ($CDCl_3$): δ (ppm) = 9.97 (s, 1 H, –CHO), 9.88 (s, 1 H, –CHO), 7.85 (dd, J_1 = 8.5 Hz, J_2 = 2.2 Hz, 1 H, Ar-H), 7.59, (d, J = 1.9 Hz, 1 H, Ar-H), 7.40 (combined

signals, 2 H, Ar-H), 7.25 (d, J = 7.3 Hz, 1 H, Ar-H), 7.20 (d, J = 8.5 Hz, 1 H, Ar-H), 7.17 (dd, J_1 = 8.5 Hz, J_2 = 2.2 Hz, 1 H, Ar-H), 7.03 (d, J = 8.5 Hz, 1 H, Ar-H), 7.03 (d, J = 2.2 Hz, 1 H, Ar-H), 6.94 (d, J = 2.5 Hz, 1 H, Ar-H), 6.87 (dd, J_1 = 8.5 Hz, J_2 = 2.5 Hz, 1 H, Ar-H), 6.80 (d, J = 8.5 Hz, 1 H, Ar-H), 6.36 (d, J = 12.0 Hz, 1 H, CH=CH, *cis*), 6.23 (d, J = 12.0 Hz, 1 H, CH=CH, *cis*), 3.83 (s, 3 H, $–OCH_3$), 3.74 (s, 3 H, $–OCH_3$), 3.72 (s, 3 H, $–OCH_3$), 3.71 (s, 3 H, $–OCH_3$).

^{13}C NMR ($CDCl_3$): δ (ppm) = 191.96, 190.90 (2-CHO), 162.10, 159.02, 157.57, 156.15, 138.39, 136.96, 136.71, 133.35, 132.05, 131.92, 131.28, 131.20, 129.72, 129.61, 129.51, 129.38, 129.28, 128.50, 128.19, 126.11, 124.01,114.38, 113.10, 110.81, 110.64, 109.01, 55.88, 55.72, 55.45, 55.23.

(*E*)-5'-[2-(4'-Formyl-4,2'-dimethoxybiphenyl-2-yl)-vinyl]-6,2'-dimethoxybiphenyl-3-carbaldehyde (30)

The *cis*-stilbene **29** (200 mg. 0.39 mmol) was dissolved in anhydrous THF (10 mL), diphenyl disulfide (17.2 mg, 78.6 µmol) was added and the mixture was heated to reflux for 24 h. Additional Ph–S–S–Ph (11.0 mg, 50.0 µmol) was added and heating was continued for 2 h. The solvent was removed *in vacuo* and the residue was purified by CC (silica gel, EtOAc/*n*-hexane 1:1); pale yellow solid, 170 mg (85%), mp 72–76 °C; HPLC: Nucleosil 100-5, 4.0 × 250 mm, EtOAc/*n*-hexane 30:70.

IR: ν (cm^{-1}) = 3001, 2936, 2834, 2727, 2359, 2342, 1683 (C=O), 1595, 1573, 1499, 1485, 1460, 1414, 1384, 1264, 1247, 1230, 1195, 1178, 1165, 1150, 1120, 1021, 1000, 958, 809, 736.

^{1}H NMR ($CDCl_3$): δ (ppm) = 10.01 (s, 1 H, –CHO), 9.90 (s, 1 H, –CHO), 7.87 (dd, J_1 = 8.5 Hz, J_2 = 2.2 Hz, 1 H, Ar-H), 7.75 (d, J = 2.2 Hz, 1 H, Ar-H), 7.50 (combined signals, 2 H, Ar-H), 7.36 (d, J = 7.6 Hz, 1 H, Ar-H), 7.31 (dd, J_1 = 8.5 Hz, J_2 = 2.2 Hz,

1 H, Ar-H), 7.27 (d, *J* = 2.5 Hz, 1 H, Ar-H), 7.19 (combined signals, 2 H, Ar-H), 7.05 (d, *J* = 8.5 Hz, 1 H, Ar-H), 6.98 (d, *J* = 16.1 Hz, 1 H, *trans*-C<u>H</u>=CH), 6.90 (d, *J* = 8.8 Hz, 1 H, Ar-H), 6.89 (dd, J_1 = 8.5 Hz, J_2 = 2.5 Hz, 1 H, Ar-H), 6.71 (d, *J* = 16.1 Hz, 1 H, *trans*-CH=C<u>H</u>), 3.90 (s, 3 H, $–OCH_3$), 3.83 (s, 3 H, $–OCH_3$), 3.81 (s, 3 H, $–OCH_3$), 3.75 (s, 3 H, $–OCH_3$).

^{13}C NMR ($CDCl_3$): δ (ppm) = 191.98, 190.91 (2-CHO), 162.08, 159.50, 157.69, 156.73, 137.45, 136.92, 136.66, 133.10, 132.66, 131.54, 129.99, 129.82, 129.58, 129.16, 128.98, 128.22, 127.80, 127.13, 126.52, 125.48, 124.16, 113.10, 111.29, 110.91, 110.02, 109.27, 55.98, 55.85, 55.81, 55.34.

MS (CI, 120 eV): *m/z* (%) = 508 (100, M^+), 374 (10), 324 (3), 286 (8), 272 (20), 270 (25), 255 (28), 239 (5), 225 (3), 155 (10).

Preparation of $TiCl_3(DME)_2$

Titanium(III) chloride (25.0 g, 16.2 mmol) was suspended in dimethoxyethane (350 mL) under argon atmosphere and heated to reflux for 2 d. After cooling, the precipitate was filtered off under argon and was washed with *n*-pentane (50 mL) and dried *in vacuo*. The product (blue crystals) was used for the following McMurry protocols.

Macrocycle 7 ((*Z*)-stilbene + tolane bridges)

To $TiCl_3(DME)_2$ (513 mg, 1.52 mmol) in anhydrous dimethoxyethane (50 mL) under argon atmosphere was added zinc dust (367 mg, 5.61 mmol) and the mixture was heated to reflux for 2 h. The tolane dialdehyde **28** (86.0 mg, 170 µmol) in anhydrous dimethoxyethane (50 mL) was added dropwise within 3 h and the mixture was heated again to reflux for 16 h. The mixture was cooled, 6 M HCl (100 mL) was added and

extracted with Et_2O (3 × 100 mL). The combined organic layers were washed with H_2O to pH 7 (~100 mL), dried ($MgSO_4$) and concentrated. The crude product was purified by CC (silica gel, CH_2Cl_2) yielding a colorless solid; 40 mg (50%); mp 120 °C; HPLC: Nucleosil 100-5, 4.0 × 250 mm, EtOAc/*n*-hexane 20:80.

IR: ν (cm^{-1}) = 2999, 2927, 2832, 2196 (–C≡C–), 1724, 1595, 1555, 1502, 1460, 1408, 1311, 1266, 1246, 1179, 1125, 1104, 1024, 965, 906, 867, 809, 730, 709, 658, 609.

^{1}H NMR ($CDCl_3$, 25 °C): δ (ppm) = 7.35 (not resolved, 1 H, Ar-H), 7.30 (not resolved, 0.50 H, Ar-H), 7.25–7.08 (combined signals, 2.70 H, Ar-H; incl. dd, J_1 = 8.5 Hz, J_2 = 2.2 Hz), 7.03 (d, *J* = 1.9 Hz, 1.50 H, Ar-H), 6.98–6.82 (combined signals, 5.00 H, Ar-H), 6.78 (d, *J* = 8.5 Hz, 1.20 H, Ar-H), 6.62 (not resolved, 2 H, *cis*-C<u>H</u>=C<u>H</u>), 3.84 (s, 3 H, $–OCH_3$), 3.83 (s, 3 H, $–OCH_3$), 3.81 (s, 3 H, $–OCH_3$) 3.71 + 3.53 (2 s, 1.42 + 1.58 H, $–OCH_3$, signals of two conformers).

^{13}C NMR ($CDCl_3$, 25 °C): δ (ppm) = 158.45, 158.41, 157.02, 156.38, 141.31, 139.44, 135.78, 134.69, 132.15, 130.89, 129.57, 129.03, 128.80, 128.72, 128.13, 126.69, 125.52, 115.83, 114.60, 114.56, 114.54, 114.52, 114.50, 114.48, 114.42, 114.32, 114.30, 114.29, 114.27, 114.26, 114.23, 114.20, 114.19, 114.15, 114.13, 114.11, 114.09, 114.08, 111.10, 111.05, 111.04, 111.98, 91.85 (–<u>C</u>≡C–), 91.83 (–C≡<u>C</u>–), 55.82, 55.75, 55.33 (2 signals).

^{1}H NMR ([D_6]-DMSO, **25 °C**): δ (ppm) = 7.31 (d, *J* = 7.6 Hz, 1 H, Ar-H), 7.23 (combined signals, 1.50 H, Ar-H; incl. dd, J_1 = 8.5 Hz, J_2 = 2.2 Hz), 7.17–7.08 (combined signals, 2.40 H, Ar-H; incl. d, *J* = 2.2 Hz), 7.02 (combined signals, 2.40 H, Ar-H), 6.95 (combined signals, 2.40 H, Ar-H; incl. d, *J* = 8.5 Hz), 6.90–6.73 (combined signals, 2.30 H, Ar-H), 6.68 (d, *J* = 12.0 Hz, 1 H, *cis*-C<u>H</u>=CH), 6.62 (d, *J* =

12.0 Hz, 1 H, *cis*-CH=C<u>H</u>), 3.80 (s, 3 H, –OCH_3), 3.77 (s, 3 H, –OCH_3), 3.76 (s, 3 H, –OCH_3), 3.67 + 3.41 (2 s_{br}, 1.80 H + 1.20 H, –OCH_3, signals of two conformers).

^{1}H NMR ([D_6]-DMSO, **75 °C**): δ (ppm) = 7.30 (dd, J_1 = 8.5 Hz, J_2 = 2.1 Hz, 1 H, Ar-H), 7.21 (dd, J_1 = 8.5 Hz, J_2 = 2.1 Hz, 1 H, Ar-H), 7.18 (combined signals, 2 H, Ar-H), 7.13 (d, J = 8.5 Hz, 1 H, Ar-H), 7.03 (combined signals, 2 H, Ar-H), 6.95 (dd, J_1 = 8.2 Hz, J_2 = 2.7 Hz, 1 H, Ar-H), 6.94 (d, J = 8.5 Hz, 1 H, Ar-H), 6.90–6.86 (combined signals, 2 H, Ar-H), 6.82 (d, J = 1.8 Hz, 1 H, Ar-H), 6.68 (d, J = 11.9 Hz, 1 H, *cis*-C<u>H</u>=CH), 6.64 (d, J = 11.9 Hz, 1 H, *cis*-CH=C<u>H</u>), 3.82 (s, 3 H, –OCH_3), 3.79 (s, 3 H, –OCH_3), 3.78 (s, 3 H, –OCH_3), 3.56 (s, 3 H, –OCH_3).

The rotation barrier was approximated to ~ 70 kJ/mol (see calculation method for compound **3**).

^{13}C NMR ([D_6]-DMSO, **75 °C**): δ (ppm) = 157.94, 157.02, 156.79 (broad), 155.97, 139.85, 138.89, 134.28, 133.24, 131.35, 131.23, 129.14, 128.99, 128.54, 128.44, 128.27 (br), 128.13, 127.41, 126.01, 124.39, 123.19, 119.93 (br), 114.42, 114.04, 113.89, 111.71, 111.59, 111.44 (broad), 92.25 (–<u>C</u>≡C–), 91.44 (–C≡<u>C</u>–), 55.45, 55.31, 55.11 (broad), 54.97.

MS (CI, 120 eV): *m/z* (%) = 474 (53, M^+), 473 (100), 472 (49), 459 (21), 458 (26), 443 (4), 442 (36), 421 (5), 414 (9), 384 (18), 342 (3), 324 (3), 198 (7), 197 (12), 179 (4).

Macrocycle 5 (two (*Z*)-stilbene bridges)

To $TiCl_3(DME)_2$ (513 mg, 1.52 mmol) in anhydrous dimethoxyethane (50 mL) under argon atmosphere was added zinc dust (367 mg, 5.61 mmol) and the mixture was heated to reflux for 2 h. The (*Z*)-stilbene dialdehyde **29** (90.0 mg, 177 µmol) in anhydrous dimethoxyethane (50 mL) was added dropwise within 3 h and the mixture was heated again to reflux for 16 h. The mixture was cooled, 6 M HCl (100 mL) was

added and extracted with Et_2O (3 × 100 mL). The combined organic layers were washed with H_2O to pH 7 (~100 mL), dried ($MgSO_4$) and concentrated. The crude product was purified by CC (silica gel, CH_2Cl_2) and furthermore by preparative HPLC (Lichrospher 100 diol, 10 × 250 mm, EtOAc/*n*-hexane 10:90) yielding a colorless solid; 30 mg (36%); mp 105 °C; HPLC: Nucleosil 100-5, 4.0 × 250 mm, EtOAc/*n*-hexane 20:80.

IR: ν (cm^{-1}) = 2998, 2929, 2832, 1722, 1598, 1566, 1548, 1500, 1460, 1409, 1386, 1313, 1264, 1244, 1172, 1125, 1105, 1028, 999, 962, 903, 866, 841, 813, 795, 758.

^{1}H NMR ($CDCl_3$, 25 °C): δ (ppm) = 7.15–7.00 (combined signals, 2 H, Ar-H; incl. 7.09 dd, J_1 = 8.5 Hz, J_2 = 2.4 Hz), 6.95–6.70 (combined signals, 7 H, 5 Ar-H, 2 *cis*-CH=CH; incl. 6.81 d, *J* = 8.5 Hz, Ar-H), 6.75 d, *J* = 11.6 Hz, *cis*-CH=CH, 6.73 d, *J* = 11.3 Hz, *cis*-CH=CH), 6.65–6.40 (combined signals, 7 H, 5 Ar-H, 2 *cis*-CH=CH; incl. 6.81 d, *J* = 2.4 Hz, Ar-H), 6.75 d, *J* = 11.6 Hz, *cis*-CH=CH), 3.87 (s, 3 H, -OCH_3), 3.84 (s, 3 H, –OCH_3), 3.67 (s, 3 H, –OCH_3), 3.89–3.80, 3.50–3.39, 3.15–3.00 (3 s, 2.10 H + 0.20 H + 0.70 H, –OCH_3, signals of two conformers).

^{13}C NMR ($CDCl_3$, 25 °C): δ (ppm) = 158.27 (br), 158.25 (br), 156.94 (br), 156.58, 155.36 (br), 139.83 (br), 138.72 (br), 138.99 (br), 138.93 (br), 138.92 (br), 138.89 (br), 138.86 (br), 138.62 (br), 138.60 (br), 132.81 (br), 132.01 (br), 131.72 (br), 131.68 (br), 131.24, 130.56, 129.65–128.56 (br), 128.25 (br), 127.78 (br), 126.60, 121.79 (br), 115.04 (br), 114.90, 112.63, 111.08, 110.36, 109.73 (br), 55.96, 55.85 (br), 55.27, 55.12 (br).

^{1}H NMR ([D_6]-DMSO, **25 °C**): δ (ppm) = 7.60–6.30 (combined signals, 16 H, 12 Ar-H and 4 *cis*-CH=CH), 3.80 (s, 3 H, –OCH_3), 3.72 (s, 3 H, –OCH_3), 3.57 (s, 3 H, –OCH_3), 3.94–3.65, 3.06–2.85 (2 s, 2.30 H + 0.70 H, –OCH_3, signals of two conformers).

^{1}H NMR ([D$_6$]-DMSO, **75 °C**): δ (ppm) = 7.12 (dd, J_1 = 8.2 Hz, J_2 = 2.4 Hz, 1 H, Ar-H), 7.09 (d, J = 8.5 Hz, 1 H, Ar-H), 6.87 (d, J = 8.5 Hz, 1 H, Ar-H), 6.86 (d, J = 2.4 Hz, 1 H, Ar-H), 6.81 (dd, J_1 = 8.5 Hz, J_2 = 2.7 Hz, 1 H, Ar-H), 6.73 (d, J = 11.6 Hz, 1 H, *cis*-CH=CH), 6.71 (d, J = 11.8 Hz, 1 H, *cis*-CH=CH), 6.74–6.59 (combined signals, 4 H, Ar-H), 6.53 (combined signals, 2 H: d, J = 11.6 Hz, 1 H, *cis*-CH=CH + 1 H, Ar-H), 6.48 (d, J = 2.4 Hz, 1 H, Ar-H), 6.44 (d, J = 12.2 Hz, 1 H, *cis*-CH=CH), 6.31 (not resolved, 1 H, Ar-H), 3.82 (s, 3 H, –OCH_3), 3.74 (s, 3 H, –OCH_3), 3.60 (s, 3 H, –OCH_3), 3.54 (s_{br}, 3 H, –OCH_3).

The rotation barrier was approximated to ~ 70 kJ/mol (see calculation method for compound **3**).

^{13}C NMR ([D$_6$]-DMSO, **75 °C**): δ (ppm) = 157.63, 156.50, 156.21, 155.08, 139.01, 137.39 (br), 132.31 (br), 132.29 (br), 131.02, 130.90, 130.26 (br), 129.78, 129.29, 128.96, 128.64, 128.50, 127.68, 126.99, 126.25, 120.20 (br), 114.53, 112.13, 111.38, 110.65, 55.41, 55.32, 54.82, 54.78.

MS (CI, 120 eV): *m/z* (%) = 476 (20, M^+), 475 (17), 447 (100), 299 (33), 261 (17), 158 (1), 135 (3), 112 (4).

Macrocycle 6 (*E* and (*Z*)-stilbene bridges)

To $TiCl_3(DME)_2$ (513 mg, 1.52 mmol) in anhydrous dimethoxyethane (50 mL) under argon atmosphere was added zinc dust (367 mg, 5.61 mmol) and the mixture was heated to reflux for 2 h. The (*E*)-stilbene dialdehyde **30** (80.0 mg, 157 μmol) in anhydrous dimethoxyethane (50 mL) was added dropwise within 3 h and the mixture was heated again to reflux for 16 h. The mixture was cooled, 6 M HCl (100 mL) was added and extracted with Et_2O (3 × 100 mL). The combined organic layers were washed with H_2O to pH 7 (~100 mL), dried ($MgSO_4$) and concentrated. The crude

product was purified by CC (silica gel, CH_2Cl_2) yielding a colorless solid; 40 mg (53%); mp 104°C; HPLC: Nucleosil 100-5, 4.0 × 250 mm, EtOAc/*n*-hexane 20:80.

IR: ν (cm^{-1}) = 2935, 2834, 2727, 2042, 1684, 1595, 1573, 1499, 1460, 1414, 1384, 1247, 1149, 1120, 1021, 1000, 958, 900, 867, 809, 781, 736.

^{1}H NMR ($CDCl_3$, 25 °C): δ (ppm) = 7.33 (d, *J* = 8.2 Hz, 0.45 H, Ar-H), 7.27 (combined signals, 0.94 H, Ar-H), 7.25–7.23 (combined signals, 1.18 H, Ar-H), 7.19 (d, *J* = 2.5 Hz, 0.69 H, Ar-H), 7.16 (d, *J* = 2.2 Hz, 0.55 H, Ar-H), 7.14 (d, *J* = 2.5 Hz, 0.55 H, Ar-H), 7.10 (d, *J* = 7.3 Hz, 0.65 H, Ar-H), 7.02 (d, *J* = 9.5 Hz, 1.65 H, Ar-H), 6.98 (not resolved, 0.58 H, Ar-H), 6.96 (d, *J* = 2.2 Hz, 0.58 H, Ar-H), 6.94 (combined signals, 0.94 H, Ar-H), 6.92 (combined signals, 1.33 H, Ar-H), 6.89–6.86 (combined signals, 1.59 H, Ar-H), 6.81 (d, *J* = 8.2 Hz, 1.20 H, Ar-H), 6.65 (d, *J* = 12.3 Hz, 0.55 H, *cis*-C$\underline{H}$=CH), 6.63 (d, *J* = 12.3 Hz, 0.45 H, *cis*-C$\underline{H}$=CH), 6.59 (d, *J* = 12.3 Hz, 1 H, *cis*-CH=C$\underline{H}$), 6.51 (d, *J* = 16.1 Hz, 0.66 H, *trans*-C$\underline{H}$=CH), 6.45 (d, *J* = 16.1 Hz, 0.46 H, *trans*-C$\underline{H}$=CH), 3.88 (s, 3 H, $–OCH_3$), 3.84 + 3.83 (2 s, 1.85 H + 1.15 H, $–OCH_3$, signals of two conformers), 3.80 (s, 3 H, $–OCH_3$), 3.72 + 3.44 (2 s, 1.39 H + 1.61 H, $–OCH_3$, signals of two conformers).

^{13}C NMR ($CDCl_3$, 25 °C): δ (ppm) = 159.18, 159.07, 157.64, 156.89, 156.41, 156.37, 139.20, 138.86, 136.98, 136.07, 133.99, 133.68, 133.63, 133.47, 133.12, 133.08, 132.80, 132.52, 132.28, 131.67, 131.40, 131.10, 130.97, 130.88, 130.66, 130.14, 129.82, 129.65, 129.47, 129.40, 129.30, 129.26, 128.80, 128.55, 128.45, 128.38, 127.97, 127.85, 127.80, 127.00, 126.85, 126.55, 126.59, 125.55, 121.13, 120.02, 113.23, 112.98, 111.60, 110.96, 110.89, 110.73, 110.56, 110.35, 108.44, 108.38, 55.91, 55.85, 55.69, 55.67, 55.60, 55.43, 55.27 (2 signals).

^{1}H NMR ([D_6]-DMSO, **25 °C**): δ (ppm) = 7.34–7.27 (combined signals, 2 H, Ar-H), 7.24–7.17 (combined signals, 2.50 H, Ar-H), 7.15–7.09 (combined signals, 1 H, Ar-

H), 7.06–7.01 (combined signals, 1.50 H, Ar-H), 6.98–6.94 (combined signals, 2 H, Ar-H), 6.91 (d, *J* = 8.5 Hz, 1 H, Ar-H), 6.89–6.78 (combined signals, 3 H, Ar-H), 6.66 (d, *J* = 12.5 Hz, 1 H, *cis*-C<u>H</u>=CH), 6.63 (d, *J* = 12.2 Hz, 1 H, *cis*-CH=C<u>H</u>), 6.34 (d, *J* = 16.1 Hz, 0.60 H, *trans*-C<u>H</u>=CH), 6.30 (d, *J* = 16.1 Hz, 0.40 H, *trans*-CH=C<u>H</u>), 3.83 (s, 3 H, $–OCH_3$), 3.77 + 3.75 (2 s, 1.70 H + 1.30 H, $–OCH_3$, signals of two conformers), 3.72 (s, 3 H, $–OCH_3$), 3.66 + 3.34 (2 s, 1.50 H + 1.50 H, $–OCH_3$, signals of two conformers).

^{1}H NMR ([D$_6$]-DMSO, **75 °C**): δ (ppm) = 7.30 (dd, J_1 = 8.2 Hz, J_2 = 2.1 Hz, 1 H, Ar-H), 7.27 (d, *J* = 1.5 Hz, 1 H, Ar-H), 7.22 (d, *J* = 8.2 Hz, 1 H, Ar-H), 7.17 (dd, J_1 = 8.2 Hz, J_2 = 2.4 Hz, 1 H, Ar-H), 7.08 (d, *J* = 16.5 Hz, 1 H, *trans*-C<u>H</u>=CH), 7.06–7.00 (not resolved, 1 H, Ar-H), 7.04 (d, *J* = 8.5 Hz, 1 H, Ar-H), 7.02 (d, *J* = 2.1 Hz, 1 H, Ar-H), 6.91 (d, *J* = 8.5 Hz, 1 H, Ar-H), 6.88 (dd, J_1 = 8.2 Hz, J_2 = 2.7 Hz, 1 H, Ar-H), 6.85 (d, *J* = 2.4 Hz, 1 H, Ar-H), 7.00–6.82 (not resolved, 2 H, Ar-H), 6.68 (d, *J* = 12.2 Hz, 1 H, *cis*-C<u>H</u>=CH), 6.63 (d, *J* = 12.2 Hz, 1 H, *cis*-CH=C<u>H</u>), 6.40 (d, *J* = 16.1 Hz, 1 H, *trans*-C<u>H</u>=CH), 3.85 (s, 3 H, $–OCH_3$), 3.78 (s, 3 H, $–OCH_3$), 3.74 (s, 3 H, $–OCH_3$), 3.65–3.30 (s_{br}, 3 H, $–OCH_3$)

The rotation barrier was approximated to ~ 70 kJ/mol (see calculation method for compound **3**).

^{13}C NMR ([D$_6$]-DMSO, **75 °C**): δ (ppm) = 158.62, 157.10, 156.52, 156.02, 136.01, 132.45, 132.38, 131.27, 130.88, 129.87, 129.32, 128.35, 128.05, 127.82, 127.55, 127.48, 126.49, 125.26, 120.86, 112.95, 111.42, 111.12, 108.06, 55.40, 55.23, 55.01 (br), 54.87.

MS (CI, 120 eV): *m/z* (%) = 476 (19, M$^+$), 475 (100), 461 (49), 444 (2), 429 (12), 324 (2), 162 (4).

5'-[2-(4'-Formyl-4,2'-dimethoxybiphenyl-2-yl)ethyl]-6,2'-dimethoxy-biphenyl-3-carbaldehyde (31)

The alkyne **28** (410 mg, 0.81 mmol) was dissolved in EtOAc (50 mL) and Lindlar catalyst (5 % Pd on $CaCO_3$, poisoned with $Pb(OAc)_2$; 344 mg, 161 µmol) as well as two drops of quinoline were added. The Parr hydrogenation apparatus was flushed several times with hydrogen and hydrogenation was performed for 5 h at 3.5 bar (~50 psi). The catalyst was filtered off and the solvent removed *in vacuo*. The crude product was purified by CC (silica gel, EtOAc/*n*-hexane 2:1) yielding a pale yellow oil which solidifies on standing; 391 mg (95%), mp 74–76 °C; HPLC: Nucleosil 100-5, 4.0 × 250 mm, EtOAc/*n*-hexane 30:70.

IR: ν (cm^{-1}) = 3001, 2935, 2834, 2728, 1684 (C=O), 1595, 1499, 1460, 1414, 1384, 1264, 1247, 1194, 1178, 1165, 1150, 1120, 1021, 1000, 958, 900, 868, 809, 736.

^{1}H NMR ($CDCl_3$): δ (ppm) = 9.96 (s, 1 H, –CHO), 9.90 (s, 1 H, –CHO), 7.84 (dd, J_1 = 8.5 Hz, J_2 = 2.2 Hz, 1 H, Ar-H), 7.66 (d, J = 2.2 Hz, 1 H, Ar-H), 7.45–7.46 (combined signals, 2 H, Ar-H), 7.24 (d, J = 7.6 Hz, 1 H, Ar-H), 7.07 (d, J = 8.2 Hz, 1 H, Ar-H), 7.04 (d, J = 8.5 Hz, 1 H, Ar-H), 6.88 (dd, J_1 = 8.5 Hz, J_2 = 2.2 Hz, 1 H, Ar-H), 6.84–6.85 (combined signals, 2 H, Ar-H), 6.79 (d, J = 8.5 Hz, 1 H, Ar-H), 6.73 (d, J = 2.2 Hz, 1 H, Ar-H), 3.83 (s, 6 H, –OCH_3), 3.82 (s, 3 H, –OCH_3), 3.71 (s, 3 H, –OCH_3), 2.67 (combined signals, 4 H, CH_2CH_2).

^{13}C NMR ($CDCl_3$): δ (ppm) = 191.9, 190.9 (2-CHO), 162.1, 159.3, 157.6, 155.2, 141.5, 137.4, 136.90, 133.6, 132.9, 132.1, 131.5, 131.1, 130.8, 129.6, 128.7, 126.2, 124.0, 114.6, 111.3, 111.0, 110.8, 109.0, 55.9, 55.8, 55.7, 55.2, 36.3, 36.0 ($2CH_2$).

Tetramethylether of Isoplagiochin C (3)

To $TiCl_3(DME)_2$ (513 mg, 1.52 mmol) in anhydrous dimethoxyethane (50 mL) under argon atmosphere was added zinc dust (367 mg, 5.61 mmol) and the mixture was heated to reflux for 2 h. The bibenzyl dialdehyde **31** (94.0 mg, 185 µmol) in anhydrous dimethoxyethane (50 mL) was added dropwise within 3 h and the mixture was heated again to reflux for 16 h. The mixture was cooled, 6 M HCl (100 mL) was added and extracted with Et_2O (3 × 100 mL). The combined organic layers were washed with H_2O to pH 7 (~100 mL), dried ($MgSO_4$) and concentrated. The crude product was purified by CC (silica gel, CH_2Cl_2) yielding a colorless oil which solidifies; 50 mg (56%); HPLC: Nucleosil 100-5, 4.0 × 250 mm, EtOAc/*n*-hexane 20:80.

IR: ν (cm^{-1}) = 2905, 1560, 1535, 1460, 1425, 1390, 1335, 1270, 1210, 1160, 1110, 1060, 1035, 1030, 1000, 960, 865, 815.

^{1}H NMR ($CDCl_3$, 25 °C): δ (ppm) = 7.21 (not resolved, 1 H, Ar-H), 7.14 (not resolved, 1 H, Ar-H), 7.08 (not resolved, 1 H, Ar-H), 7.05 (not resolved, 1 H, Ar-H), 6.98 (not resolved, 1 H, Ar-H), 6.90–6.86 (combined signals, 3 H, Ar-H), 6.84, (d, *J* = 9.8 Hz, 1 H, Ar-H), 6.81 (dd, J_1 = 8.2 Hz, J_2 = 2.8 Hz, 1 H, Ar-H), 6.75 (d, *J* = 8.2 Hz, 1 H, Ar-H), 6.68 (2 d, *J* = 12.0 Hz, 1 H, *cis*-C<u>H</u>=CH), 6.61 (2 d, *J* = 12.0 Hz, 1 H, *cis*-CH=C<u>H</u>), 6.50 (not resolved, 1 H, Ar-H), 3.85 (s, 3 H, $–OCH_3$), 3.81 + 3.80 (2 s, 3 H, $–OCH_3$, signals of two conformers), 3.68 + 3.50 (2 s, 3 H, $–OCH_3$, signals of two conformers), 2.90–2.31 (m, 4 H, $–CH_2CH_2–$).

^{13}C NMR ($CDCl_3$, 25 °C): δ (ppm) = 158.99, 157.62, 157.02, 156.24, 156.22, 155.28, 155.25, 143.66, 143.18, 140.07, 139.36, 135.78, 135.72, 135.54, 133.91, 133.86, 133.76, 133.74, 132.08, 131.41, 131.21, 131.02, 130.51, 130.28, 130.25, 130.08, 129.70, 129.65, 129.55, 129.17, 129.01, 128.97, 128.90, 128.88, 128.70, 128.67, 127.69, 127.55, 127.26, 126.99, 125.52, 120.79, 114.14, 113.90, 111.46, 111.00,

110.97, 110.78, 110.62, 110.59, 110.52, 110.39, 55.88, 55.76, 55.64, 55.59, 55.52, 55.39, 55.15, 53.41, 38.34, 37.89, 37.56, 36.84.
